# Advances and Prospects in MOF-Based Platforms for Tumor Hyperthermia

**DOI:** 10.3390/bioengineering13060693

**Published:** 2026-06-17

**Authors:** Diyi Feng, Liqin Ge

**Affiliations:** State Key Laboratory of Digital Medicine Engineering, School of Biological Science and Medical Engineering, Southeast University, Nanjing 211189, China; fdy1017117943@163.com

**Keywords:** MOFs, tumor hyperthermia, photothermal therapy, microwave hyperthermia, magnetic hyperthermia, combination therapy

## Abstract

Metal-organic framework (MOF)-based materials have become promising platforms for tumor hyperthermia by integrating energy conversion, tumor microenvironment regulation, and multimodal therapy within programmable porous structures. This review summarizes recent advances in intrinsic MOFs, MOF composites, and MOF-derived materials for photothermal therapy, microwave hyperthermia, and magnetic hyperthermia. The reviewed studies show that high-valence metal MOFs mainly provide stable and modifiable frameworks, whereas transition-metal, magnetic, and multimetallic MOFs contribute to redox regulation, ROS generation, magnetic response, and microwave energy dissipation. Beyond localized heat generation, MOF-based platforms enhance therapeutic efficacy by combining hyperthermia with chemotherapy, chemodynamic therapy, metabolic intervention, immunotherapy, and imaging guidance. These integrated strategies help overcome incomplete ablation, thermotolerance, oxidative stress resistance, and tumor recurrence. However, clinical translation is still limited by insufficient standardization, uncertain degradation behavior, metal-ion safety, and inadequate thermal dose control. Future development should emphasize mechanism-oriented design, controllable composition, long-term biosafety, and image-guided thermal regulation to advance MOF-based hyperthermia toward precise and clinically relevant cancer therapy.

## 1. Introduction

Malignant tumors remain a major global health challenge [1,2]. Surgery, radiotherapy, and chemotherapy have achieved considerable clinical progress, yet their efficacy remains constrained by tumor heterogeneity [3], invasiveness and metastatic potential [4], therapeutic resistance [5], and the complex tumor microenvironment (TME) [6]. Conventional treatments may also damage normal tissues during tumor eradication [7] and remain insufficient to prevent recurrence [8], distant metastasis [9], and drug resistance [10]. Therefore, new therapeutic strategies that combine high tumor targeting [11], low systemic toxicity [12], and synergistic multimodal efficacy [13] are urgently needed.

Tumor hyperthermia has emerged as an important treatment modality that applies external physical energy to elevate local tissue temperature, disrupting tumor homeostasis and inhibiting growth [14]. Common energy sources, including near-infrared light (NIR) [15], alternating magnetic field (AMF) [16], and microwave (MW) [17], can be converted into heat and preferentially deposited within tumor regions under appropriate energy delivery and material localization conditions, thereby increasing the local temperature to damage tumor cells or suppress tumor growth [18,19]. According to the therapeutic temperature and mode of action, tumor hyperthermia can generally be divided into high-temperature thermal ablation and mild hyperthermia [20,21]. High-temperature thermal ablation is usually performed above 55 °C [22] and rapidly induces coagulative necrosis, enabling the effective eradication of localized tumors [23]. By contrast, mild hyperthermia is maintained at 39–45 °C [24] and exerts its antitumor effects mainly by inducing cellular stress injury [25], disrupting protein homeostasis [26], impairing mitochondrial function [27], promoting apoptosis [28], and modulating immune responses [29]. These features make mild hyperthermia particularly suitable for combination with drug delivery [30], immunotherapy (IT) [31], and TME regulation [32]. Compared with conventional therapies, hyperthermia allows external regulation of energy input and can be combined with other modalities, but its therapeutic benefit depends strongly on spatial heat deposition, tissue penetration, and thermal dose control [33,34]. In addition to directly inducing thermal damage in tumor cells, hyperthermia can increase tumor cell membrane permeability [35], facilitate drug accumulation and cellular uptake [36], enhance tumor sensitivity to radiotherapy (RT) and chemotherapy (CT) [37], and induce immunogenic cell death [38]. Nevertheless, hyperthermia monotherapy faces several limitations, such as heterogeneous heat distribution [39], insufficient tumor selectivity [40], thermal injury to normal tissues [41], and cellular thermotolerance [42]. Therefore, improving selective energy deposition in tumors [43], enhancing thermal conversion efficiency [44], reducing thermal damage to normal tissues [45], and integrating hyperthermia with other modalities remain key challenges.

Metal-organic frameworks (MOFs) are porous hybrid materials constructed from metal ions or clusters coordinated with organic ligands [46,47]. The high specific surface area [48], tunable pore structures [49], and responsiveness to the TME [50] of MOFs enable precise design of both composition and function. MOF-based compounds possess potential as photothermal agents [51], microwave-responsive sensitizers [52], and magnetothermal agents [53], while also serving as carriers for therapeutic or diagnostic components [54]. Their structural features allow the integration of multiple treatment modalities and intelligent therapy platforms, enabling combined energy conversion [55], drug delivery [56], TME modulation [57], and imaging-guided therapy [58].

Based on structural composition and functional origin, MOF-based thermal therapeutic materials are categorized as intrinsic MOFs, MOF composites, and MOF-derived materials. Intrinsic MOFs rely on their metal nodes, organic ligands, or metal-ligand interactions to generate photothermal [59], magnetic [60], or microwave-responsive properties [61]. MOF composites incorporate exogenous functional components, such as photothermal agents [62], magnetic nanoparticles [63], or MW sensitizers [64], thereby improving energy conversion efficiency and expanding therapeutic functions. MOF-derived materials, produced by pyrolysis [65], carbonization [66], sulfidation [67], or oxidation [68], retain the porous architecture and morphology of the parent MOFs while improving photothermal efficiency, magnetic response, or MW absorption [69,70]. In summary, these design strategies elevate MOF-based systems beyond passive drug carriers, allowing MOF-based systems to move beyond passive delivery toward platforms in which energy conversion, drug release, and microenvironmental responses can be co-designed.

**Figure 1:**
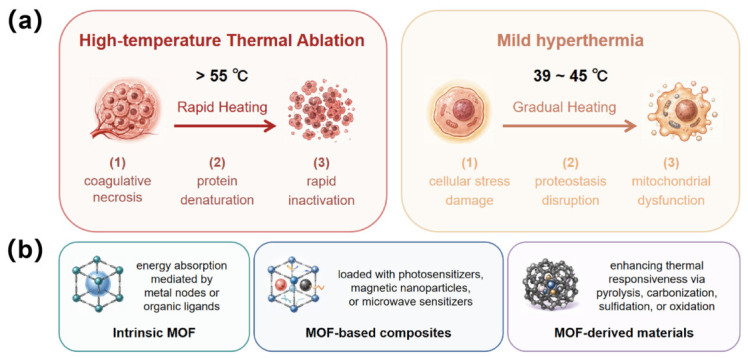
The classification of tumor hyperthermia and mechanisms of MOF-based compounds in hyperthermia. (**a**) High-temperature thermal ablation and mild hyperthermia classified by treatment temperature and mode of action. (**b**) Main mechanisms by which MOF-based compounds participate in tumor hyperthermia.

Although several recent reviews have summarized MOF-based phototherapy, drug delivery, or cancer theranostics, most of them focus primarily on photothermal or photodynamic treatment, general nanomedicine applications, or isolated therapeutic mechanisms. In contrast, this review summarizes recent progress in MOF-based compounds for tumor hyperthermia (Figure 1), highlighting structural features, energy conversion mechanisms, applications in photothermal therapy (PTT), microwave hyperthermia (MWH), and magnetic hyperthermia (MHT), as well as combination strategies and intelligent responsive platforms. By organizing recent studies, according to high-valence metal MOFs, transition-metal MOFs, magnetic MOFs, and multimetallic MOFs, this review provides a materials-oriented framework for understanding how structural stability, redox activity, dielectric loss, magnetic response, ROS generation, tumor microenvironment regulation, and imaging capability can be integrated into MOF-based hyperthermia platforms. Rather than treating hyperthermia as a single heat-generating modality, we emphasize its combination with CT, chemodynamic therapy (CDT), metabolic intervention, IT, and imaging-guided treatment. This perspective distinguishes the present review from previous summaries and provides a more systematic basis for the rational design and translational evaluation of multifunctional MOF-based tumor hyperthermia systems.

## 2. Structural Features and Classification of MOF-Based Compounds

The functionality of MOF-based compounds is dictated by the coordinated contribution of metal nodes and organic ligands [71,72]. Metal ions or clusters define the coordination stability [73], crystalline architecture [74], and degradation profile of the framework [75], while also endowing the material with redox activity [76], catalytic capability [77], magnetic responsiveness [78], and imaging potential [79]. Organic ligands complement these functions by modulating optical absorption [80] and hydrophilic/hydrophobic properties [81]. Therefore, in tumor hyperthermia, MOFs should not be regarded merely as conventional drug carriers. Instead, they represent structurally programmable multifunctional platforms in which the selection of metal nodes and organic ligands, together with pore-structure regulation and surface modification, can be rationally exploited to achieve energy responsiveness [82], thermal conversion [83], catalytic therapy [84], controlled drug release [85], and imaging diagnosis [86].

Compared with conventional inorganic nanoparticles or polymeric carriers, MOFs are distinguished by their highly programmable structural composition [87,88]. Metal nodes can impart microwave responsiveness [89], magnetic responsiveness [90], Fenton-like catalytic activity [91], glutathione-depleting capacity [92], and magnetic resonance imaging capability [93]. High-valence metal nodes generally form robust metal-oxygen coordination networks, making them suitable for constructing stable MOF scaffolds with tunable functionalization, drug-loading capacity, and imaging compatibility [94]. In contrast, transition-metal nodes such as Fe, Cu, and Mn can participate in valence-state cycling, thereby enabling Fenton or Fenton-like catalysis, GSH depletion, ROS amplification, and metal-dependent regulated cell death. These features allow hyperthermia to be integrated with catalytic therapy, redox modulation, and TME regulation [95]. Multimetallic nodes can further improve microwave absorption and catalytic activity through electronic coupling, interfacial polarization, and cooperative redox cycling. However, the increased compositional complexity also raises additional challenges related to metal distribution control, ion leakage, and long-term biosafety evaluation. The organic ligands can contribute to light absorption [96] and photosensitization [97]. Porphyrins, phthalocyanines, HHTP, conductive polymers, and other π-conjugated components can strengthen near-infrared absorption and promote photothermal conversion through non-radiative relaxation. The incorporation of exogenous photothermal agents such as ICG, IR780, CuS, and PPy further compensates for the intrinsically weak absorption, limited photostability, or insufficient thermal conversion capability of some MOF frameworks [98]. In addition, the internal pore architecture of MOFs facilitates the loading of therapeutic and functional components, including chemotherapeutic drugs [99], photosensitizers [100], ionic liquids [101], and immunomodulatory molecules [102]. These structural features allow MOF-based compounds to be tailored to the specific requirements of different hyperthermia modalities. For photothermal therapy, material design mainly emphasizes near-infrared absorption [103] and efficient non-radiative relaxation [104]. However, improved photothermal performance cannot be attributed solely to stronger optical absorption. It is also governed by the spatial dispersion of photothermal agents within the MOF pores, aggregation-induced quenching, interfacial heat transfer, drug retention, and heating stability during repeated irradiation cycles [105]. For microwave hyperthermia, dielectric loss [106], ionic conduction [107], and polarization relaxation [108] are more critical determinants of microwave-to-heat conversion. Ionic liquids, polar molecules, and multimetallic nodes can increase MW energy dissipation by promoting ionic conduction, dipolar relaxation, interfacial polarization, and dielectric loss [109]. For magnetic hyperthermia, heat generation depends on magnetic loss [110] and relaxation processes [111] of magnetic components under an alternating magnetic field. For magnetic components such as Fe_3_O_4_, FeAu, and ferrites, magnetothermal conversion under an alternating magnetic field is governed by particle size, magnetic anisotropy, saturation magnetization, and spatial distribution [112]. Accordingly, small molecules and functional components should not be considered as isolated additives. Rather, they modulate overall hyperthermia performance by reshaping the electronic structure, energy dissipation pathways, pore microenvironment, and interfacial heat-transfer behavior.

Particle size, morphology, and colloidal stability are critical determinants of the in vivo behavior of MOF-based hyperthermia platforms. Oversized particles may show limited deep tumor penetration and impaired clearance, whereas excessively small particles may suffer from reduced drug-loading capacity, shortened circulation stability, or rapid renal elimination [113]. Morphological engineering, including hollow architectures, core-shell structures, nanosheets, and porous carbon-derived frameworks, can alter specific surface area, mass transport, thermal diffusion pathways, and drug-release behavior, but it may also increase synthetic complexity and batch-to-batch variability [114]. Biodegradability and clearance should therefore be considered integral elements of MOF design rather than post hoc safety evaluations. Controlled degradation of MOF frameworks in acidic tumor microenvironments or lysosomes can facilitate drug release, metal-ion-mediated catalysis, and imaging-signal activation. However, premature framework dissociation during blood circulation may cause drug leakage, nonspecific metal-ion release, and systemic toxicity, whereas overly stable or poorly degradable MOFs may accumulate in the liver, spleen, and reticuloendothelial system, increasing the risk of chronic inflammation and organ toxicity [115]. An ideal MOF-based hyperthermia platform should therefore maintain sufficient stability during circulation, respond selectively within the TME or intracellular environment, and be gradually eliminated after treatment through degradation, metabolism, or excretion.

Therefore, this chapter focuses on the structural features of MOF-based compounds and discusses how different metal nodes influence framework stability, energy responsiveness, catalytic activity, imaging performance, and in vivo degradation behavior (Table 1). This discussion provides a materials-level basis for understanding the subsequent applications of MOF-based platforms in photothermal therapy, microwave hyperthermia, magnetic hyperthermia, and intelligent stimuli-responsive therapeutic systems.

### 2.1. High-Valence Metal MOFs

**Figure 2:**
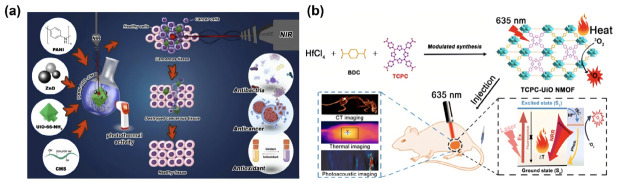
Representative high-valence metal MOF-based platforms for tumor hyperthermia. (**a**) UiO-66-NH_2_-based photothermal platform in which the stable Zr-MOF scaffold supports functional integration, enhanced NIR responsiveness, localized heat generation, and tumor cell damage [127]; (**b**) Hf-based TCPC-UiO NMOF constructed from HfCl^4^, BDC, and TCPC for imaging-guided PTT, where porphyrinic units mediate light absorption, heat generation, and ROS production, while the Hf framework enables CT, thermal, and photoacoustic imaging [133].

High-valence metal MOFs are typically constructed from metal nodes with high charge density, such as Zr^4+^, Ti^4+^, and Hf^4+^ [116,117]. These metal ions behave as hard Lewis acids and readily coordinate with carboxylate, phosphonate, or phenolic hydroxyl ligands to form strong metal–oxygen coordination bonds. As a result, the corresponding frameworks generally exhibit high structural stability and favorable hydrothermal resistance [118]. Representative examples include the UiO series [119], MIL-125 [120], PCN-type frameworks [121], and porphyrin-based MOFs [122]. In tumor hyperthermia, the main value of these MOFs does not usually arise from strong intrinsic catalytic activity of the metal nodes. Instead, it lies in their robust frameworks, tunable pore structures, and broad post-synthetic modification capacity, which make them structural scaffolds for constructing hyperthermia systems in which stability and functional loading can be systematically tuned (Figure 2).

Zr-based MOFs exhibit high structural stability [123], which can help prevent premature framework disintegration during blood circulation. For this reason, they have been widely used as carriers for photosensitizers [124], chemotherapeutic agents [125], and immunomodulatory molecules [126]. UiO-66, UiO-67, and their amino or carboxyl-functionalized derivatives possess well-defined pore structures and abundant sites for surface modification, making them suitable for constructing pH-responsive, GSH-responsive, or ligand-targeted hyperthermia platforms [127]. In addition, Zr-based porphyrinic MOFs can immobilize porphyrin photosensitizing units in an ordered framework, thereby reducing aggregation-induced quenching and improving the efficiency of photodynamic or photothermal therapy [128]. For mild hyperthermia, the stable and chemically modifiable structure of Zr-based MOFs is particularly advantageous for controlled drug release and multimodal therapeutic synergy. Ti-based MOFs and their derivatives are of particular interest in photoresponsive and catalytic therapies [129]. Represented by MIL-125, Ti-based MOFs exhibit semiconductor-like characteristics, and Ti–O clusters can participate in photoinduced electron-transfer processes [130]. Through defect engineering, oxygen-vacancy regulation, metal doping, or carbonization, the band gap of these materials can be narrowed and additional defect levels can be introduced, thereby extending their optical response into the near-infrared region [131]. Although TiO_2_ itself responds strongly to ultraviolet light, its near-infrared responsiveness is limited. Therefore, MOF-derived strategies for constructing TiO_2−x_, C/TiO_2_ composites, or metal oxide heterojunctions represent important approaches to improving photothermal conversion and photocatalytic activity [132]. In tumor hyperthermia, these materials can integrate photothermal effects with ROS generation, enabling synergistic PTT and photodynamic therapy (PDT). Hf-based MOFs share coordination-chemical similarities with Zr-based MOFs, but the higher atomic number of Hf gives them additional advantages in radiotherapy sensitization, computed tomography imaging, and PDT [133]. Hf-based porphyrinic MOFs can function simultaneously as photosensitizing platforms and radiosensitizers, thereby enhancing energy deposition and ROS generation in combination therapy systems [134]. Although Hf-based MOFs have been less extensively explored in standalone hyperthermia than Zr and Ti-based systems, their high stability, high atomic number, and ligand-design flexibility provide considerable potential for imaging-guided hyperthermia and radiotherapy-hyperthermia combination therapy.

Overall, high-valence metal MOFs are mainly valued for their structural robustness, tunable porosity, and broad post-synthetic modifiability, which make them suitable for constructing well-defined and functionally controllable theranostic platforms. However, their metal nodes generally show limited intrinsic thermal conversion capability or Fenton-like catalytic activity. Therefore, additional functionalization strategies, including photosensitizing ligand incorporation, defect engineering, exogenous photothermal agent loading, or MOF-derived transformation, are often required to enhance their therapeutic performance.

### 2.2. Transition Metal MOFs

**Figure 3:**
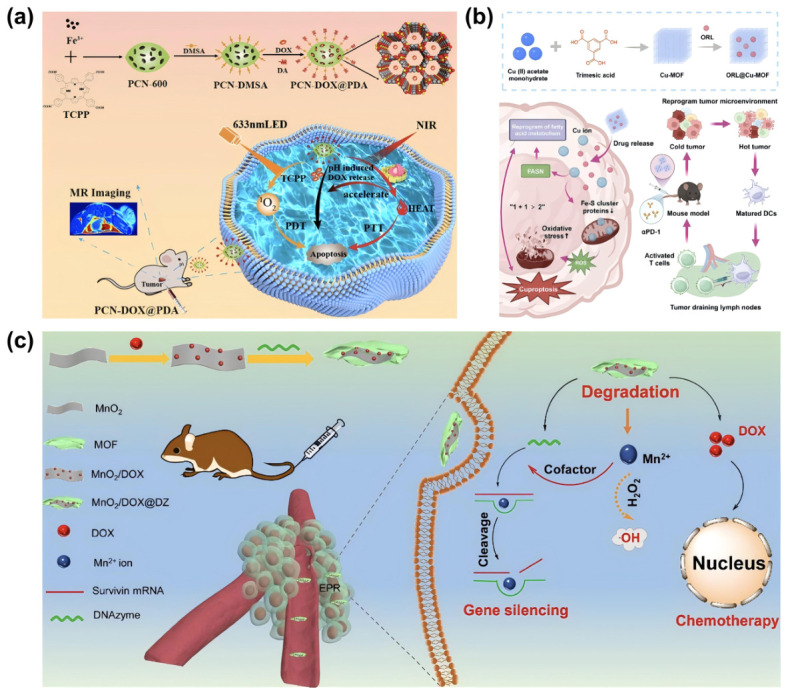
Representative transition metal MOF-based platforms for tumor hyperthermia. (**a**) PCN-DOX@PDA accelerates the Fenton reaction through local heating generated by photothermal therapy, thereby promoting ROS production and increasing the sensitivity of tumor cells to thermal stimulation [140]; (**b**) ORL@Cu-MOF enhances tumor killing by releasing Cu^2+^, disrupting copper homeostasis, and inducing mitochondrial metabolic dysfunction [143]; (**c**) MnO_2_/DOX@DZ catalyzes the conversion of H_2_O_2_ into O_2_ to alleviate tumor hypoxia, while simultaneously promoting ROS generation through Fenton-like reactions and weakening the antioxidant capacity of tumor cells [145].

Transition-metal MOFs are typically constructed from transition-metal nodes such as Fe, Cu, and Mn, and represent a widely investigated category with broad functional diversity of MOF-based materials for tumor hyperthermia [135,136]. Compared with high-valence metal MOFs, transition-metal MOFs are distinguished by the variable valence states and strong electron-transfer capabilities of their metal nodes. These characteristics enable them to participate directly in redox reactions, Fenton or Fenton-like catalysis, GSH depletion, ionic homeostasis disruption, and electromagnetic energy responses [137]. Therefore, transition-metal MOFs can serve not only as delivery matrices, but also as active therapeutic components that directly contribute to catalytic therapy, oxidative stress amplification, thermal sensitization, and TME regulation (Figure 3).

Fe-based MOFs are among the most widely used systems for integrating hyperthermia with CDT and imaging-guided treatment. The Fe^2+^/Fe^3+^ redox cycle can catalyze the conversion of excessive H_2_O_2_ in the tumor microenvironment into highly reactive ·OH, thereby inducing oxidative stress and tumor cell death [138]. In parallel, Fe-containing materials possess magnetic responsiveness, which enables their use in T_2_-weighted MRI or magnetothermal therapy-related platforms [139]. During photothermal or microwave hyperthermia, local temperature elevation can accelerate Fe-mediated Fenton-like reactions and improve ROS generation efficiency. The accumulated ROS can further disrupt antioxidant and thermotolerance-related systems, including GSH, GPX4, and HSPs, thereby increasing tumor cell sensitivity to thermal stimulation [140]. Cu-based MOFs exhibit strong redox activity and capacity to support multiple therapeutic mechanisms, including redox regulation, GSH depletion, and heat-assisted ROS generation. The Cu^+^/Cu^2+^ redox cycle can participate in Fenton-like reactions and catalyze H_2_O_2_ conversion into ·OH, while Cu^2+^ can react with GSH and weaken the antioxidant defense capacity of tumor cells [141]. Some Cu-based MOFs and their derivatives, such as CuS, Cu_2_S, and Cu/C composites, also show strong near-infrared absorption and favorable photothermal conversion efficiency, making them suitable for photothermal therapy [142]. In addition, copper ions are closely associated with cuproptosis-related mechanisms. Cu-based MOFs can release Cu^2+^ in the TME and enhance tumor killing by disturbing copper homeostasis, impairing mitochondrial metabolism, promoting lipoylated protein-associated cytotoxicity, and depleting GSH [143]. Accordingly, Cu-based MOFs have distinctive advantages in hyperthermia combined with CDT, metabolic intervention, and cuproptosis induction. Mn-based MOFs combine redox regulation, hypoxia relief, and imaging capability. Mn^2+^ can serve as a T_1_-weighted MRI contrast agent, allowing tumor localization before treatment and monitoring during therapeutic intervention [144]. MnO_2_ can respond to excessive H_2_O_2_ in the TME to generate O_2_, thereby alleviating tumor hypoxia and enhancing PDT or ROS-related therapeutic effects. It can also consume GSH and weaken the antioxidant capacity of tumor cells [145]. For microwave hyperthermia, Mn-related nodes can enhance polarization loss and microwave absorption, thereby improving local microwave-to-heat conversion efficiency [146]. Therefore, Mn-based MOFs are frequently used to construct microenvironment-responsive, imaging-guided, and ROS-amplifying hyperthermia platforms. Co and Ni-based MOFs are particularly valuable for electromagnetic response and MOF-derived material construction. Co-based MOFs, such as ZIF-67, can serve as precursors for preparing Co/C, Co_3_O_4_/C, or CoS_x_ materials with enhanced microwave absorption, photothermal conversion, or catalytic performance [147]. Co nodes can also participate in GSH-responsive degradation, H_2_O_2_ catalysis, and redox regulation [148]. Ni-based MOFs and their derivatives are commonly used to construct conductive carbon-based or sulfide composite materials, which can improve photothermal performance and electromagnetic loss capacity [149].

Overall, the major advantage of transition metal-based MOFs is that their metal nodes can directly participate in therapeutic processes, including catalytic reactions, electron transfer, redox regulation, GSH depletion, imaging enhancement, and electromagnetic energy response. These properties make them particularly suitable for constructing multimodal synergistic therapeutic platforms. Nevertheless, the safety concerns associated with transition metal ion release are also more pronounced. Future studies should therefore carefully balance therapeutic activity with biosafety, especially in terms of metal dose, release kinetics, tissue accumulation, and long-term clearance.

### 2.3. Magnetic Metal-Based MOFs

**Figure 4:**
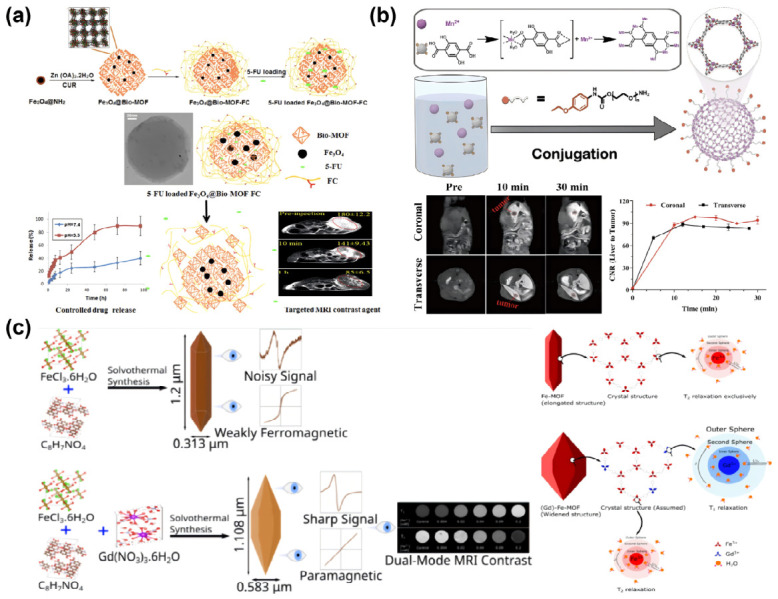
Representative magnetic MOF-based platforms with imaging capability. (**a**) Fe_3_O_4_-loaded Bio-MOF-Fc integrates 5-FU delivery with magnetic responsiveness, enabling pH-responsive drug release and T_2_-weighted MRI-guided tumor localization [154]; (**b**) Mn-based MOF provides paramagnetic T_1_-weighted MRI contrast through Mn^2+^ coordination, allowing time-dependent monitoring of tumor accumulation [155]; (**c**) Gd-doped Fe-MOF improves MRI performance by modulating magnetic properties and enhancing contrast, demonstrating the value of metal-node engineering for image-guided MOF-based hyperthermia [156].

Magnetic metal-based MOFs and magnetic MOF composites are important material systems for magnetic hyperthermia and microwave hyperthermia [150]. Magnetic hyperthermia relies on the conversion of magnetic energy into heat by magnetic components under an alternating magnetic field, mainly through hysteresis loss, Néel relaxation, and Brownian relaxation [151]. Microwave hyperthermia, in contrast, can enhance energy deposition through magnetic loss, dielectric loss, and interfacial polarization [152]. Therefore, MOFs containing magnetic metal nodes such as Fe, Mn, and Co, as well as MOF composites loaded with Fe_3_O_4_, FeAu alloys, or ferrite nanoparticles, are relevant to deep-tumor hyperthermia because magnetic and microwave fields can penetrate deeper than NIR light, although heat distribution and field safety remain critical constraints (Figure 4).

Magnetic metal nodes can be directly incorporated into MOF frameworks to endow the materials with intrinsic magnetic responsiveness. For example, Fe-based MOFs can combine magnetic response with Fenton-like catalytic activity, Mn-based MOFs can integrate MRI capability with redox regulation, and Co-based MOFs or their derivatives can enhance magnetic loss or microwave absorption. These materials offer the advantage of structural integrity and relatively homogeneous distribution of metal nodes, which facilitates the coupling of hyperthermia, catalytic therapy, and imaging functions within a single framework. However, their magnetothermal conversion efficiency is often constrained by the crystalline structure of the MOF and the coordination environment of the metal nodes. Therefore, practical magnetic hyperthermia systems frequently require further integration with magnetic nanoparticles. Magnetic nanoparticles can also be immobilized within the pores, shells, or surfaces of MOFs to construct core–shell or embedded magnetic MOF composites. Fe_3_O_4_ nanoparticles possess favorable biocompatibility and well-established magnetothermal performance. However, free Fe_3_O_4_ nanoparticles are prone to aggregation, which can reduce magnetic responsiveness, produce heterogeneous local thermal fields, and lead to poorly controlled in vivo distribution [153]. The MOF framework can provide a confinement and dispersion effect, helping magnetic particles maintain small sizes and uniform spatial distribution, thereby improving the stability and reproducibility of magnetothermal conversion. Meanwhile, the porous structure of MOFs can accommodate functional molecules such as DOX, 5-FU, AIPH, or immunomodulators, enabling thermally triggered drug release or radical generation after heating under an alternating magnetic field. Another important advantage of magnetic MOF systems is their compatibility with imaging monitoring. Fe_3_O_4_ and Fe-based MOFs can provide T_2_-weighted MRI contrast [154], Mn-based MOFs can generate T_1_-weighted MRI signals [155], and Gd-doped or Gd-based MOFs can further enhance T_1_ imaging capability [156]. These features allow magnetic hyperthermia to evolve from a purely heat-based intervention into an imaging-guided precision therapeutic modality. MRI can be used to monitor material accumulation within tumors and determine the optimal timing for alternating magnetic field exposure. In addition, thermal imaging or MRI-based thermometry can help optimize therapeutic dosing and reduce damage to adjacent normal tissues [157]. Therefore, the key objective of magnetic MOF design is not limited to improving magnetothermal conversion efficiency, but also includes controlling the size, spatial distribution, metabolic fate, and imaging traceability of magnetic components.

### 2.4. Multimetallic MOFs

**Figure 5:**
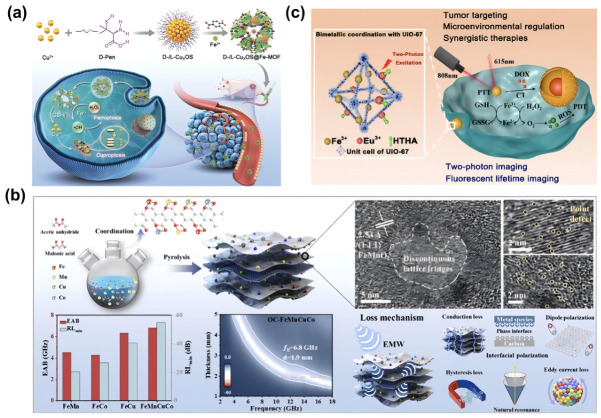
Representative multimetallic MOF-based platforms for tumor hyperthermia and combination therapy. (**a**) D−/L−CuxOS@Fe−MOF establishes a mutually reinforcing redox cycle between copper and iron ions, thereby enhancing ROS generation at the tumor site [161]; (**b**) OC-FeMnCuCo improves microwave absorption by enhancing dielectric loss, interfacial polarization, and dipolar relaxation [163]; (**c**) CIDF enables dual-modal imaging-guided combination therapy involving PTT, PDT, and CT, thereby achieving precise tumor cell killing [165].

In recent years, multimetallic MOFs have emerged as an important direction in the design of MOF-based hyperthermia materials. The single metal node usually provides a limited range of functions, Fe nodes can contribute to catalytic therapy and imaging [158], Cu nodes can mediate redox regulation and photothermal conversion [159], and Mn nodes can alleviate hypoxia while enabling MRI contrast [160]. By incorporating multiple metal nodes into the MOF framework, multimetallic MOFs can integrate complementary functions related to structural stability, energy responsiveness, catalytic therapy, diagnostic imaging, and tumor microenvironment regulation. This compositional integration provides a rational strategy for constructing multifunctional hyperthermia platforms with coordinated therapeutic and imaging capabilities (Figure 5).

Multimetallic MOFs can enhance electron transfer and catalytic efficiency. Different metal centers with variable valence states can form intraframework electron-transfer pathways, facilitating Fe^2+^/Fe^3+^, Cu^+^/Cu^2+^, or Mn^2+^/Mn^3+^/Mn^4+^ redox cycling and thereby improving Fenton or Fenton-like reaction efficiency [161]. In the tumor microenvironment, multimetallic nodes can synergistically catalyze H_2_O_2_ conversion into ·OH while simultaneously consuming GSH and weakening the antioxidant barrier [162]. This cooperative effect is particularly important for mild hyperthermia, where the direct cytotoxicity of moderate temperature elevation is limited and therefore requires amplification by ROS generation, lipid peroxidation, or regulated cell death pathways. Multimetallic MOFs can also increase electromagnetic loss through additional polarization centers, interfacial charge redistribution, or magnetic loss pathways. In microwave hyperthermia, they can enhance microwave absorption by increasing dielectric loss, interfacial polarization, and dipolar relaxation [163]. Differences in charge distribution among distinct metal nodes can generate additional polarization centers, leading to stronger energy dissipation under a microwave field. When magnetic metals such as Fe, Mn, or Co are incorporated, magnetic loss can be further introduced to improve microwave-to-heat conversion efficiency [164]. In MHT, the combination of multimetallic or alloy nanoparticles with MOF frameworks can also enhance saturation magnetization and improve the stability of magnetothermal conversion. Multimetallic doping further facilitates the integration of therapeutic and imaging functions [165]. For example, Gd/Fe bimetallic MOFs can provide T_1_/T_2_ dual-modal MRI signals while enhancing microwave hyperthermia or ROS generation. Mn/Cu bimetallic MOFs can combine Mn-mediated hypoxia relief with Cu-mediated GSH depletion and Fenton-like reactions. Fe/Cu bimetallic MOFs can strengthen microwave absorption and CDT through dual-metal redox cycling. These examples illustrate that multimetallic MOFs are not simply mixtures of different metal components; rather, their value lies in the rational coupling of complementary metal-dependent functions within a unified framework.

The construction of multimetallic MOFs also introduces new challenges. Different metal ions vary in coordination ability, reaction kinetics, and hydrolytic stability, which may lead to structural heterogeneity, reduced batch-to-batch reproducibility, or difficulty in precisely controlling metal distribution. In addition, the pharmacokinetics and long-term toxicity associated with the release of multiple metal ions are more complex than those of single-metal systems. Therefore, the design of multimetallic MOFs should not simply pursue an increasing number of metal components. Instead, metal combinations should be selected according to clearly defined therapeutic objectives. For microwave hyperthermia, priority can be given to metal combinations that enhance dielectric loss and magnetic loss. For CDT, metals with complementary redox cycling capacity are more appropriate. For imaging-guided therapy, imaging-relevant nodes such as Gd, Mn, or Fe can be introduced. Overall, multimetallic MOFs represent an important direction in the evolution of MOF-based hyperthermia materials from single-function systems toward synergistically integrated platforms. The key value lies in improving antitumor responses in reported models through electronic coupling, catalytic complementarity, and imaging synergy among different metal nodes. Future development, however, should place greater emphasis on compositional controllability and long-term safety, so as to avoid increasing the difficulty of clinical translation through excessive structural complexity.

This metal-node-based classification underscores that different metal nodes endow MOF materials with distinct structural stability, energy responsiveness, and biological functionality. High-valence metal MOFs are more suitable for constructing stable and chemically modifiable frameworks. Transition metal MOFs can directly participate in catalytic therapy and redox regulation through valence-state changes and electron transfer. Magnetic MOFs and their composites are particularly useful for magnetic hyperthermia, microwave sensitization, and imaging guidance. Multimetallic MOFs further integrate energy conversion, catalytic therapy, and diagnostic imaging through cooperative interactions among metal nodes. In this sense, metal nodes not only determine the structural characteristics of MOF-based compounds, but also largely define which hyperthermia modality they are best suited for and which therapeutic functions they can integrate.

**Table 1:** Major Categories of MOF-Based Hyperthermia Platforms: Metal Nodes and Functional Features.

MOF Category	Representative Metal Nodes	Structural and Functional Features	Advantages	Limitations
High valence metal MOFs	Zr, Ti, Hf based MOFs,	Strong metal-oxygen coordination, high framework stability, tunable porosity, and abundant sites for post-synthetic modification	High structural robustness, favorable chemical stability, good modifiability, and controllable pore architecture	Intrinsic thermal conversion and catalytic activity are often limited, additional photothermal agents, defect engineering, ligand design, or MOF derived transformation are usually required
Transition metal MOFs	Fe, Cu, Mn, Co, Ni based MOFs	Variable valence states, strong electron transfer capability, redox activity, Fenton or Fenton-like catalysis, GSH depletion, and TME responsiveness	Functionally active metal nodes enable catalytic therapy, redox regulation, TME modulation, and thermal sensitization within one framework	Potential metal ion leakage, off-target redox toxicity, tissue accumulation, and long-term clearance risks require careful biosafety evaluation
Magnetic metal MOFs	Fe, Mn, Co based MOFs	Magnetic responsiveness, magnetic loss, hysteresis loss, Néel relaxation, Brownian relaxation, and compatibility with MRI	Enable deep tissue heating, magnetic targeting or monitoring, reduced dependence on optical penetration, and potential MRI guidance	Magnetothermal efficiency depends on particle size, magnetic anisotropy, saturation magnetization, aggregation state, and field parameters
Multimetallic MOFs	Fe Cu, Mn Cu, Gd Fe, and other bimetallic or polymetallic MOFs	Multiple metal nodes integrated into one framework, intermetallic charge transfer, enhanced polarization, cooperative redox cycling, and multifunctional imaging or catalytic capability	Integrate complementary metal dependent functions, improve energy conversion and catalytic efficiency, and support synergistic therapy within a unified platform	More difficult to control metal distribution, coordination chemistry, batch reproducibility, pharmacokinetics, and Multi-ion toxicity, excessive compositional complexity may hinder clinical translation

## 3. MOF-Based Platforms for Tumor Hyperthermia

**Figure 6:**
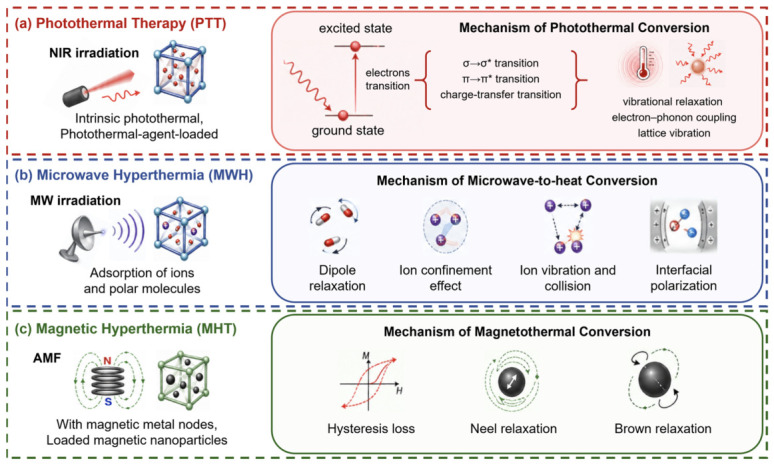
Classification and mechanisms of MOF-based tumor hyperthermia. (**a**) PTT and the photothermal conversion mechanism; (**b**) MWH and the microwave-to-heat conversion mechanism; (**c**) MHT and the magnetothermal conversion mechanism.

As shown in Figure 6, MOF-based tumor hyperthermia can be broadly divided into three major modalities according to the external energy source and heat-generation mechanism: photothermal therapy (PTT) [166], microwave hyperthermia (MWH) [167], and magnetic hyperthermia (MHT) [168]. From the mechanistic perspective, MOF-based hyperthermia extends beyond direct energy-to-heat conversion and should be viewed as a sequential therapeutic process involving energy harvesting, material-specific energy dissipation, localized thermal deposition, and downstream biological amplification. The dominant energy-conversion pathway differs substantially among hyperthermia modalities. In photothermal therapy, therapeutic heating is mainly determined by NIR light absorption and non-radiative relaxation. In microwave hyperthermia, energy deposition is governed by dielectric loss, ionic conduction, interfacial polarization, and dipolar relaxation. Magnetic hyperthermia, by contrast, relies on the ability of magnetic components to dissipate energy under an alternating magnetic field through hysteresis loss, Néel relaxation, and Brownian relaxation. These differences indicate that MOF-based hyperthermia platforms should be engineered according to the specific energy-transfer requirements of each modality, rather than being designed merely as generic nanocarriers. In this context, MOFs should be considered not merely as passive carriers, but as structurally programmable therapeutic platforms whose composition, porosity, electronic structure, and functional interfaces can be tailored to optimize energy harvesting, thermal conversion, and biological therapeutic output. This section summarizes these modalities and discusses how MOF-based materials improve energy conversion efficiency, enhance tumor-localized energy deposition, and increase therapeutic efficacy through structural design and functional integration (Table 2).

### 3.1. MOF-Mediated PTT

PTT represents one of the most explored applications of MOF-based materials in tumor hyperthermia [169]. Upon near-infrared light (NIR) irradiation, electrons in the photothermal agents transition from the ground state to an excited state [170]. Instead of being released through radiative processes such as fluorescence or phosphorescence, the energy is predominantly converted into heat via non-radiative mechanisms, including vibrational relaxation, electron-phonon coupling, and interfacial thermal transfer [171]. The generated heat raises the temperature locally within the tumor, thereby producing a therapeutic thermal effect. The efficiency of this process depends not only on how strongly the material absorbs light, but also on how the absorbed excitation energy is dissipated and transferred as heat. Therefore, effective photothermal agents are generally expected to combine strong near-infrared absorption with suppressed radiative decay and efficient non-radiative relaxation.

MOFs are particularly well suited for PTT due to their highly tunable structural and compositional features. The metal nodes, organic ligands, pore sizes, and overall morphology can be precisely tailored [172], enabling the design of platforms with strong NIR absorption. The high surface area and ordered pore networks further allow MOFs to carry photothermal agents such as indocyanine green (ICG) [173], IR780 [174], and Au nanoparticles [175], mitigating issues such as aggregation, photobleaching, poor stability, and short in vivo retention. MOFs can also be transformed into MOF-derived materials through carbonization, sulfurization, oxidation, or defect engineering, which enhances NIR absorption, photothermal conversion efficiency, and thermal stability [176]. MOF-based PTT platforms can be classified according to the origin and engineering strategy of their photothermal function into intrinsic photothermal MOFs, composite photothermal MOFs, and MOF-derived photothermal materials. In intrinsic photothermal MOFs, light harvesting and heat generation are encoded within the framework itself, typically through the electronic structures of the metal nodes, conjugated ligands, or metal-ligand interactions. Composite photothermal MOFs rely on the integration of external photothermal agents, where the MOF matrix improves the dispersion, stability, tumor accumulation, and combinational loading capacity of these agents. MOF-derived photothermal materials are produced by converting parent MOFs into carbonaceous, sulfide, oxide, or hybrid nanostructures, which often exhibit broader optical absorption, improved charge transport, and enhanced thermal stability. In summary, intrinsic MOFs emphasize framework-level photothermal activity, composite MOFs focus on functional integration, and MOF-derived materials exploit structural inheritance and compositional transformation.

In MOF-based PTT, photothermal performance is often compared using photothermal conversion efficiency, maximum temperature elevation, or heating rate. These parameters, however, are highly sensitive to the experimental configuration. Reported photothermal conversion efficiencies are not always directly comparable across studies because laser power density, irradiation time, material concentration, sample volume, optical path, heat-loss correction, and thermal boundary conditions are frequently not standardized. In many in vitro studies, marked temperature increases are achieved by using relatively high power densities or prolonged irradiation, conditions that may not be readily transferable to in vivo treatment or clinical practice. Excessive laser input may not only overestimate the intrinsic contribution of the material to heat generation but also induce nonspecific heating of normal tissues, skin burns, or local inflammatory responses [177]. Interpretation of in vivo photothermal data also requires careful consideration of temperature measurement. Tumor surface temperature, central temperature, and marginal temperature should be distinguished, since infrared thermal imaging mainly reflects surface heating and cannot reliably determine whether deeper tumor regions have reached an effective thermal dose [178]. A more rigorous assessment would combine infrared thermal imaging with multipoint temperature measurements and heat-transfer modeling, while also monitoring temperature changes at the tumor margin and in adjacent normal tissues. Such information is essential for evaluating not only heating efficiency but also spatial thermal distribution and safety margins. The distinction between NIR-I and NIR-II photothermal systems should not be reduced to penetration depth alone. NIR-I lasers, such as 808 nm irradiation, benefit from mature instrumentation, broad availability of photothermal agents, and operational convenience, but their relatively stronger tissue scattering and absorption make them more suitable for superficial or locally accessible tumors. NIR-II lasers, such as 1064 nm irradiation, generally exhibit lower tissue scattering and greater penetration potential, making them attractive for deeper or larger solid tumors [179]. This advantage, however, imposes stricter requirements on material design, including strong NIR-II absorption, efficient heat generation at clinically acceptable power densities, and long-term biosafety [180]. Thus, the choice between NIR-I and NIR-II systems should be guided by tumor location, tissue depth, material absorption characteristics, external energy safety thresholds, and therapeutic objectives, rather than by wavelength range alone. For future MOF-based photothermal platforms, standardized, reproducible, and clinically relevant performance evaluation will be more informative than simply pursuing higher temperature elevation or higher photothermal conversion efficiency.

**Figure 7:**
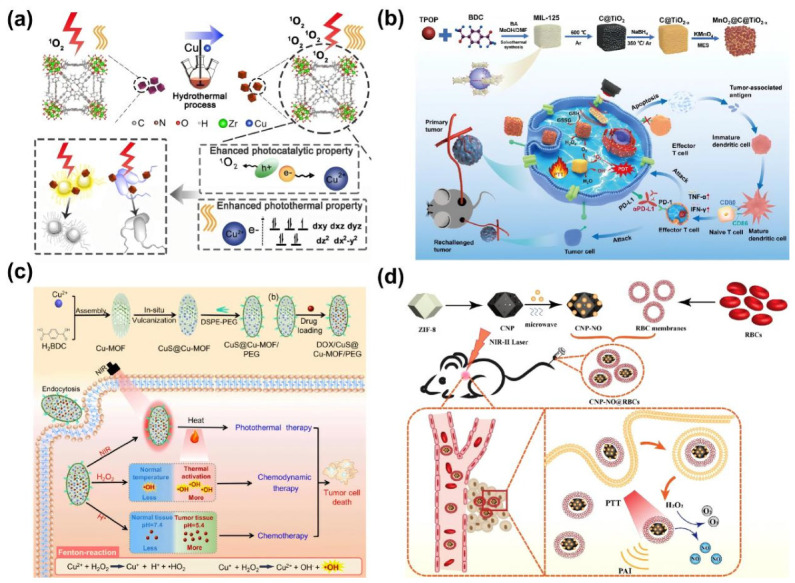
Representative strategies to enhance photothermal performance in MOF−based systems. (**a**) Incorporation of copper ions into a porphyrin-based MOF introduced d−d transitions, promoting light absorption and photothermal conversion [184]; (**b**) The MnO_2_@C@TiO_2−x_ nanoplatform achieved band-gap narrowing through oxygen vacancy formation, expanding the spectral response range [187]; (**c**) CuS@Cu-MOF nanocomposites employed an in situ sulfurization strategy to ensure uniform CuS distribution, resulting in improved photothermal conversion efficiency [191]; (**d**) Porous carbon nanoparticles with RBC membrane coating (CNP-NO@RBCs) combined high-temperature carbonization and acid etching to preserve porosity while enhancing photothermal conversion efficiency [197].

Intrinsic photothermal MOFs generate NIR absorption primarily through metal nodes, organic ligands, or electronic interactions between them [181]. Organic ligands with extended π-conjugated structures, such as porphyrins, phthalocyanines, and hexahydroxytriphenylene, enhance light absorption via π-π electronic transitions and convert absorbed photons into heat through non-radiative relaxation [182]. Metal nodes with variable valence states further extend the absorption range in the NIR region through mechanisms including d-d transitions, metal-ligand charge transfer, intervalence charge transfer, or free-carrier absorption [183]. Han et al. engineered a Cu-metalated porphyrinic Zr-MOF based on PCN-224 to improve its photocatalytic and photothermal performance. By coordinating Cu^2+^ within the porphyrin centers, the electronic structure of the framework was modulated to promote charge separation and electron transfer, thereby enhancing ROS generation. The Cu^2+^ sites also introduced d–d transition pathways, which strengthened light absorption and favored non-radiative heat dissipation. Among the prepared formulations, Cu_10_MOF exhibited the most efficient photothermal response, reaching 48.4 °C within 5 min of irradiation and retaining stable heating performance during repeated light on/off cycles [184] (Figure 7a). Material band structure and defect states also critically influence photothermal performance [185]. Wide band-gap materials typically show limited NIR absorption. Introducing oxygen vacancies, heteroatom dopants, or valence-state modulation can reduce the band gap and create defect levels, broadening the spectral response [186]. Jiang et al. used MIL-125 as a precursor to prepare the MnO_2_@C@TiO_2−x_ nanoplatform through a one-step solid-state pyrolysis strategy. The oxygen vacancies reduced the band gap of TiO_2_ from 3.14 to 2.81 eV, extending light absorption to the 808 and 1064 nm NIR regions. Meanwhile, the MnO_2_ component simultaneously responded to H_2_O_2_ in the TME to promote reactive oxygen species (ROS) generation. This design achieved both high photothermal conversion efficiency and enhanced ROS production, enabling synergistic PTT and PDT, and exemplifies a strategy for MOF-derived materials in combination therapy [187] (Figure 7b).

Composite MOF-based photothermal systems exploit the tunable pore structure and surface functionalization of MOFs to incorporate exogenous photothermal agents, compensating for the limited intrinsic NIR absorption of some MOFs [188]. MOFs improve dispersibility of the photothermal agents in aqueous and physiological environments, mitigating aggregation-induced quenching and maintaining photothermal performance [189]. In addition, the porous architecture and surface modifications of MOFs enhance retention at tumor sites, improving local therapeutic efficacy [190]. Geng et al. reported a CuS-integrated Cu-MOF nanocomposite by using porous Cu-MOF as both the structural template and copper source for partial in situ sulfidation, followed by PEG modification to improve its biological applicability. In this architecture, CuS nanodots were generated and confined on the surface or within the pores of the Cu-MOF framework, which strengthened NIR absorption while retaining the porous structure required for drug loading. Owing to the plasmonic photothermal effect of CuS, CuS@Cu-MOF/PEG exhibited efficient heating under 1064 nm laser irradiation. The material also produced pronounced photothermal cytotoxicity against 4T1 cells, reducing cell viability to 15.3% after 8 min of NIR exposure [191] (Figure 7c).

In composite material design, coating with a photothermal layer is also a common strategy for enhancing photothermal performance [192]. Liu et al. introduced a polypyrrole (PPy) layer onto Cr-MIL-101-NH_2_ via polymer encapsulation, producing ODA@MOF/PPy composite phase-change materials. The abundant π-conjugated electrons in PPy enabled broad-spectrum light absorption across the UV-Vis-NIR range, while non-radiative relaxation converted absorbed energy into heat. The PPy coating also enhanced interfacial heat transfer, leading to higher photothermal conversion and thermal energy storage efficiency [193]. Fan et al. used MIL101-NH_2_ as the core carrier to construct an ICG-CpG@MOF nanoplatform by conjugating ICG functional units through amide bonds and further loading the immunoadjuvant CpG via porous adsorption and electrostatic interactions. This system exploited the porous structure and surface amino groups of the MOF to integrate ICG and CpG within a single platform. Under 808 nm laser irradiation, ICG-CpG@MOF showed concentration and power-dependent temperature elevation, together with maintained heating performance over repeated irradiation cycles. Meanwhile, ICG endowed the platform with near-infrared fluorescence, photoacoustic imaging, and combined photothermal/photodynamic therapeutic functions, enabling multimodal imaging-guided tumor cell killing. The CpG component further promoted immunoadjuvant release and immune activation [194]. Composite photothermal MOFs are designed to overcome several intrinsic limitations of conventional photothermal agents, including poor aqueous dispersibility, limited physiological stability, nonspecific leakage, and insufficient tumor retention. By confining photothermal components within porous frameworks or anchoring them onto modifiable surfaces, MOFs can improve their structural stability, reduce premature loss, and promote more sustained accumulation in tumor tissues. Beyond stabilizing photothermal agents, the MOF matrix also provides a versatile compartment for delivering chemotherapeutic drugs, photosensitizers, gas donors, or other therapeutic modules. This allows heat generation to be spatially coupled with complementary antitumor mechanisms, thereby strengthening local therapeutic responses when heat generation is spatially coupled with drug release, ROS generation, or immune activation.

MOF-derived materials have emerged as another important approach to enhance photothermal performance [195]. These materials preserve the porous architecture and morphology of the parent MOFs while exhibiting increased light absorption, thermal stability, and electronic conductivity [196]. Li et al. developed a ZIF-8-derived porous carbon nanoplatform by carbonizing ZIF-8 at 900 °C and subsequently removing residual zinc through acid etching. The resulting carbon nanoparticles were used to load L-arginine and were further cloaked with red blood cell membranes to obtain CNP-NO@RBCs. This design combined the strong NIR-II photothermal performance of MOF-derived carbon with the biomimetic circulation advantages of RBC membrane coating. Upon 1064 nm laser irradiation, CNP-NO@RBCs generated efficient local heating and simultaneously enabled H_2_O_2_-responsive nitric oxide release in the tumor microenvironment. The platform integrated photothermal therapy, NO gas therapy, and photoacoustic imaging within a single MOF-derived theranostic system [197] (Figure 7d). Li et al. demonstrated a MOF-derived photothermal design by carbonizing MOF-5 to generate a hierarchical porous carbon/ZnO hybrid. During carbonization, the ordered distribution of Zn nodes and organic linkers in MOF-5 enabled ZnO nanoparticles to form in situ and remain highly dispersed within the porous carbon matrix. This architecture coupled the light-harvesting function of ZnO with the broadband absorption and heat-conversion capability of porous carbon, illustrating how MOF precursors can be transformed into integrated semiconductor/carbon photothermal materials [198]. MOF-derived photothermal materials offer a distinct route to enhancing light-to-heat conversion by transforming crystalline frameworks into conductive carbonaceous, sulfide, oxide, or hybrid nanostructures. Compared with the corresponding pristine MOFs, these derivatives often display broader optical absorption and more efficient charge transport, which are favorable for photothermal energy conversion. Porous carbon derivatives can strengthen NIR responsiveness through free-carrier absorption, defect-associated electronic states, and extended π-conjugated domains, whereas metal sulfides may contribute additional photothermal activity through localized surface plasmon resonance or narrow-band-gap absorption. A key advantage of MOF-derived systems is that the precursor framework preorganizes metal nodes and organic linkers at the nanoscale, allowing the derived materials to retain, at least in part, the morphology and porosity of the original MOF. This structural inheritance enables MOF-derived photothermal platforms to integrate strong light absorption, high surface area, and drug-loading capacity, making them attractive for photothermal-based combination therapy.

### 3.2. MOF-Mediated MWH

MWH uses microwave electromagnetic energy, typically within the frequency range of 300 MHz to 300 GHz, to generate heat within tumor tissues [199]. Compared with light-triggered PTT, MWH offers deeper tissue penetration and more efficient volumetric heating, making it particularly attractive for deep-seated tumors [200]. In addition, microwave-induced heating is less affected by optical attenuation and is relatively less sensitive to the heat-sink effect, which may help achieve more uniform intratumoral temperature elevation [201]. These features allow MWH to complement PTT and MHT in settings where limited penetration depth or insufficient energy deposition remains a major obstacle. MWH offers an important advantage over NIR-mediated PTT because MW energy is less affected by optical scattering and absorption, allowing more effective heating of deep-seated tumors. Nevertheless, precise thermal control remains challenging in biologically heterogeneous tissues. MW deposition is strongly influenced by tissue hydration, dielectric properties, vascular perfusion, and applicator geometry, which may produce nonuniform temperature fields, including central overheating, insufficient heating at tumor margins, or collateral damage to surrounding normal tissues. Microwave-sensitizing materials have therefore been developed to increase tumor-selective MW absorption and improve local energy deposition. By enhancing microwave-to-heat conversion within the tumor, these materials can reduce the required irradiation power or exposure duration while maintaining therapeutic temperature levels, thereby improving the precision and safety of MWH.

MOF-based materials provide a multifunctional platform for improving microwave energy deposition and heat conversion. Their porous frameworks, tunable metal nodes, and organic–inorganic coordination structures allow microwave-responsive components to be incorporated at the molecular or nanoscale level [202]. The porous frameworks of MOFs enable the efficient incorporation of polar molecules [203] and endogenous or exogenous ions [204], followed by their spatial confinement within nanoscale channels. Under microwave irradiation, confined ions undergo high-frequency oscillation and collision, generating Joule heat more efficiently than freely mobile ions [205]. Polar molecules, especially water molecules, can also dissipate microwave energy through dipolar relaxation, further contributing to local heat generation [206]. Together, ion confinement and dipolar relaxation enhance microwave-to-heat conversion and promote tumor-localized hyperthermia. In addition, the MW responsiveness of MOFs can be further strengthened through electromagnetic loss mechanisms. The coordination structure formed by metal ions or metal clusters and organic ligands endows MOFs with excellent dielectric and magnetic properties. Therefore, MOFs can absorb MW energy and convert it into heat through both dielectric loss and magnetic loss [207]. For dielectric loss, metal-related electronic transitions and ligand-metal charge transfer can create polarization centers. Under a MW electric field, ionic polarization, dipolar polarization, and interfacial polarization occur, and the energy dissipated during polarization relaxation is converted into heat [208]. When magnetic metal nodes such as Fe or Mn are introduced, magnetic loss can further contribute to heat generation through magnetic domain reversal, natural resonance, hysteresis loss, and eddy-current loss [209]. Therefore, rational design of MOF composition and pore structure can enhance MW responsiveness and improve the therapeutic efficiency of MWH. In bimetallic and multimetallic MOF systems, the coexistence of distinct metal centers provides additional pathways for charge redistribution and redox cycling. Intermetallic charge-transfer interactions can generate abundant polarization sites and strengthen dielectric loss under microwave irradiation, thereby promoting more efficient microwave energy dissipation. As a result, multimetallic MOFs may achieve effective thermal conversion even at relatively low microwave power, which is particularly advantageous for reducing nonspecific heating of surrounding tissues.

**Figure 8:**
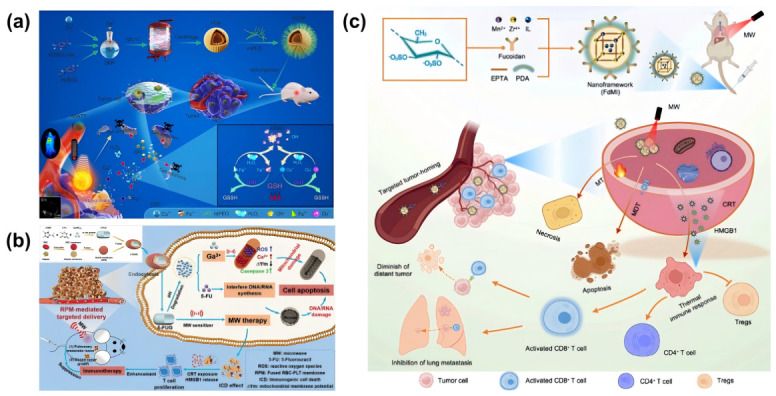
Representative MOF-based platforms for MWH. (**a**) FCMP improved MW energy absorption through Fe-Cu bimetallic electron transfer and synergistic polarization [210]; (**b**) 5-FUGR combined biomimetic membrane coating with microwave-responsive release of drugs and metal ions to enhance tumor-directed therapy [212]; (**c**) FdMI integrated ionic-liquid-mediated MW sensitization with fucoidan-assisted tumor targeting, thereby improving local MW energy deposition and therapeutic efficacy [213].

As shown in Figure 8, representative MOF-based MWH systems have been developed by integrating metal-node engineering, ion confinement, biomimetic delivery, and tumor-responsive release. Zhu et al. constructed an Fe-Cu bimetallic organic framework and further modified it with mPEG to obtain FCMP for combined MWH and CDT. The Fe-Cu framework promoted microwave energy dissipation through charge-transfer and polarization effects, leading to efficient heating under low-power MW irradiation. Meanwhile, Fe/Cu redox cycling catalyzed endogenous H_2_O_2_ to generate ROS and depleted GSH, thereby amplifying CDT-mediated oxidative damage. These combined features allowed FCMP to couple microwave sensitization with CDT-mediated oxidative damage and T_2_ MRI-guided tumor treatment [210]. Cheng et al. developed Fe-Cu MOF@PEG@SUN as a microwave-responsive therapeutic platform by integrating a Fe-Cu bimetallic MOF with sunitinib. The Fe-Cu framework enhanced microwave energy dissipation through bimetallic charge transfer and polarization effects, thereby improving local thermal conversion under microwave irradiation. In parallel, sunitinib was introduced to modulate the immunosuppressive tumor microenvironment by suppressing heat-induced PD-L1 upregulation. This integrated strategy enabled coordinated MWH, CT, and IT, resulting in effective suppression of both primary and metastatic tumors [211]. Meng et al. developed a biomimetic Ga-MOF@RPM nanoplatform, referred to as 5-FUGR, by cloaking a Ga-MOF core with the hybrid red blood cell-platelet membrane. The hybrid membrane endowed the system with prolonged blood circulation, immune evasion, and enhanced tumor accumulation, while the Ga-MOF framework served as a microwave-responsive reservoir for therapeutic drug and metal ions. Upon MW irradiation, 5-FUGR enabled localized release and thermal activation within the tumor, promoting immunogenic cell death and subsequent systemic antitumor immune activation [212]. Xu et al. developed an FdMI nanoframework by integrating Mn-doped zirconium MOFs, confined ionic liquids, and fucoidan-mediated surface functionalization. Mn incorporation and confined ionic species enhanced MW responsiveness by strengthening polarization and dielectric loss, whereas fucoidan improved tumor targeting, including accumulation in both primary tumors and metastatic lesions. Upon MW exposure, FdMI efficiently dissipated electromagnetic energy as localized heat and simultaneously promoted ROS generation through Mn-mediated catalytic activity. By coupling microwave-induced thermal ablation with ROS-amplified chemodynamic damage, this platform achieved coordinated antitumor effects against local and metastatic disease [213]. Feng et al. reported a Cu-doped Zr-MOF microwave sensitizer for liver cancer treatment by integrating MWH with microwave dynamic therapy (MWDT). The one-pot hydrothermally prepared Cu-Zr MOF exploited the ion-confining porous framework to enhance microwave-induced ionic collisions, thereby improving local microwave-to-heat conversion. In addition, Cu^2+^ sites introduced Fenton-like catalytic activity, allowing microwave-activated ·OH generation from endogenous H_2_O_2_. Under MW irradiation, the material produced both efficient thermal elevation and ROS amplification in vitro and in vivo. Further mechanistic analysis indicated that this combined treatment downregulated HSP70 and modulated the HMOX1/GPX4 axis, leading to ferroptosis-mediated tumor cell death [214]. Collectively, recent MOF-based MWH platforms have shifted from a temperature-centered design paradigm toward multifunctional systems that integrate MW sensitization, tumor-selective delivery, microenvironmental modulation, and immune activation. Metal nodes are typically engineered to enhance dielectric loss and redox catalysis, while ionic liquids (ILs) or other polar components increase MW energy dissipation through confined polarization and ion-conduction effects. Surface engineering strategies, including biomimetic membrane coating, fucoidan modification, and hyaluronic acid functionalization, further improve tumor accumulation, tissue penetration, and cellular uptake. These modular designs broaden the therapeutic role of MWH from localized thermal ablation to ROS-amplified damage and immune-mediated tumor control, thereby offering a strategy to suppress residual disease, recurrence, and metastasis.

**Figure 9:**
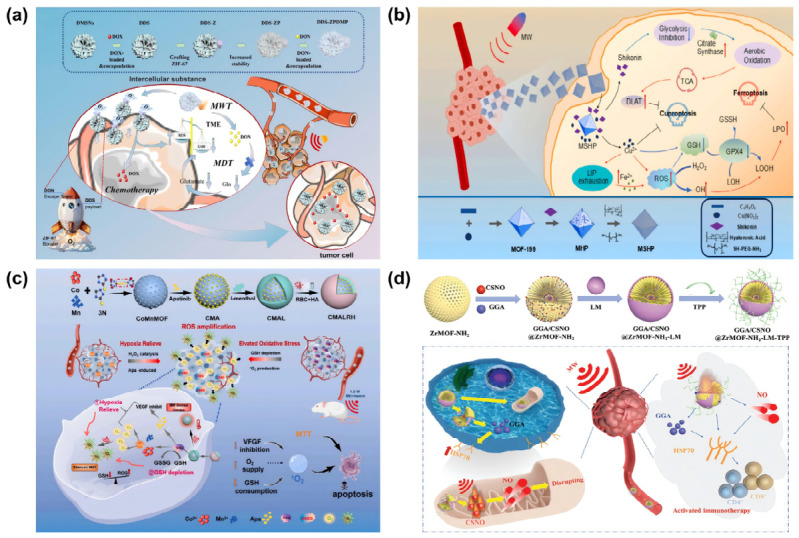
(**a**) DDS-ZPDMP utilized ZIF-67 and a Janus “nanorocket” structure to achieve microwave-triggered autonomous motion and sequential drug release into deep tumor regions [215]; (**b**) MOF-199@Shikonin@HA-PEG integrated the glycolysis inhibitor shikonin and HA-PEG modification to enhance MW responsiveness and active tumor targeting [216]; (**c**) CMALRH leveraged synergistic polarization of Co and Mn components to improve microwave-to-heat conversion efficiency [217]; (**d**) GCZMT incorporated the mitochondria-targeting ligand TPP to enable precise microwave-triggered delivery of therapeutic agents to tumor mitochondria [218].

As shown in Figure 9, several MOF-based microwave-responsive systems have been designed to improve tumor penetration, regulate the TME, and amplify microwave-mediated therapeutic effects. Zhuang et al. developed a microwave-driven Janus nanorocket, DDS-ZPDMP, by integrating a ZIF-67 propulsion module with DMSNs for DOX loading. In the TME, ZIF-67 reacted with endogenous H_2_O_2_ to generate O_2_, enabling autonomous motion under MW stimulation. The asymmetric Janus structure promoted directional movement, deeper tumor penetration, and enhanced intracellular drug accumulation, while the DMSN domain supported sustained DOX release for spatially controlled CT [215]. Liu et al. engineered MOF-199@Shikonin@HA-PEG (MSHP) by incorporating the glycolysis inhibitor shikonin into a Cu-based MOF and introducing HA-PEG for tumor-targeted delivery. Under MW irradiation, MSHP enabled the release of Cu^2+^ and shikonin within the tumor region. Shikonin inhibited glycolysis and suppressed ATP production, thereby restricting GSH biosynthesis, while Cu^2+^ catalyzed Fenton-like ROS generation and consumed residual GSH. By coupling metabolic suppression with redox disruption, MSHP weakened the antioxidant defense of tumor cells and amplified microwave-mediated therapeutic damage [216].

Wang et al. developed a TME-responsive CoMnMOF nanoplatform, CMALRH, cloaked with a red blood cell-hyaluronic acid hybrid membrane to extend circulation and enhance tumor accumulation. In the GSH-rich TME, disulfide-bond cleavage triggered framework degradation, GSH depletion, and the release of Co^2+^, Mn^2+^, and apatinib. The released Co^2+^ and Mn^2+^ catalyzed H_2_O_2_ decomposition to generate O_2_, while apatinib inhibited vascular endothelial growth factor (VEGF) expression, collectively improving tumor oxygenation. The resulting hypoxia relief promoted ROS generation and strengthened the synergy between MWH and MWDT [217]. Guo et al. developed a biomimetic mitochondria-targeted MOF platform, GCZMT, by loading an NO donor and an HSP70-regulating agent into a ZrMOF-NH_2_ framework, followed by functionalization with triphenylphosphonium (TPP). Under MW irradiation, GCZMT generated localized heat and promoted the release of bioactive components in the mitochondrial region. Mitochondria-localized thermal stress and NO release then triggered programmed cell death and immunogenic cell death, thereby amplifying systemic antitumor immunity [218]. These examples indicate that MOF-based MWH is evolving from simple thermal ablation toward multifunctional therapeutic systems that integrate deep-tumor delivery, metabolic interference, redox regulation, hypoxia relief, organelle targeting, and immune activation. The design of MOF-based microwave hyperthermia platforms is increasingly shaped by two complementary considerations: selective MW energy deposition and microwave-triggered therapeutic activation. The former relies on strategies such as multimetallic polarization, ion confinement, and active targeting to enhance intratumoral heat generation. The latter exploits microwave irradiation to activate drug release, ROS amplification, metabolic intervention, or immune modulation. Together, these strategies improve not only the spatial control of thermal deposition, but also the biological efficacy derived from a given thermal dose, thereby broadening the therapeutic potential of MOF-mediated MWH.

### 3.3. MOF-Mediated MHT

MHT employs an AMF to induce localized heating of tumor tissues through magnetic nanoparticles [219]. Under AMF stimulation, these nanoparticles convert electromagnetic energy into heat via magnetic hysteresis loss, Néel relaxation, and Brownian relaxation, allowing AMF-induced heat deposition in deeper tissues, with efficiency determined by magnetic properties, particle distribution, and field parameters without dependence on tissue dielectric properties or polar molecule content [220]. The heating efficiency and therapeutic performance are dictated by the type and magnetic characteristics of the nanoparticles. Typical materials include biocompatible ferrite nanoparticles [221], metallic magnetic nanoparticles with high magnetothermal conversion efficiency [222], and composite nanoparticles that combine enhanced heat generation with improved biocompatibility [223].

The performance of MHT is governed by a combination of magnetic parameters and biological constraints, including particle size, magnetic anisotropy, saturation magnetization, field frequency, and field amplitude. Particle size is particularly critical, small particles may generate insufficient heat because of weak magnetic relaxation, whereas larger particles are more susceptible to aggregation and may face less favorable in vivo clearance. In superparamagnetic systems, heat generation mainly arises from Néel and Brownian relaxation, while ferromagnetic or ferrimagnetic materials can additionally dissipate energy through hysteresis loss. Effective MHT materials must therefore be optimized not only for high magnetothermal conversion, but also for colloidal stability, biodistribution, clearance, and long-term biosafety. MOF-based materials offer advantages beyond serving as carriers for magnetic nanoparticles. Their highly tunable pore size, framework architecture, and specific surface area allow precise spatial control of magnetic particle distribution, mitigating aggregation-induced local overheating and ensuring uniform heat generation within tumors [224]. Furthermore, metal nodes in MOFs, particularly Fe, Mn, and Co, contribute directly to magnetic responses. Through ligand-metal charge transfer, these nodes generate local polarization and additional magnetic loss, further enhancing the conversion of magnetic energy into heat [225].

**Figure 10:**
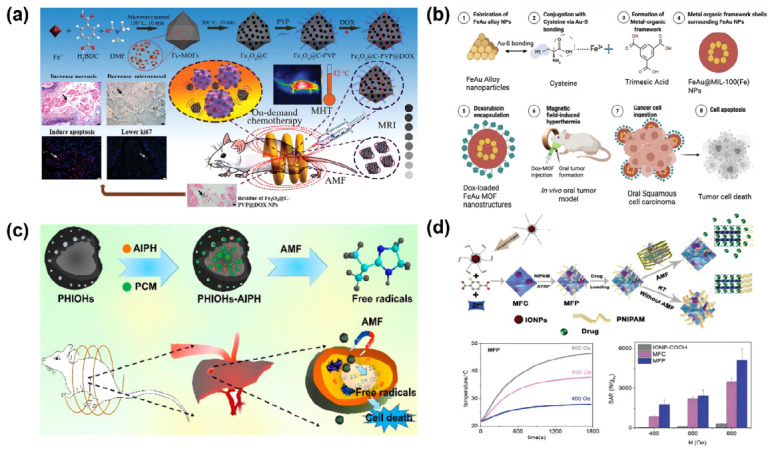
(**a**) Fe_3_O_4_@C-PVP@DOX combines T_2_-weighted MRI, AMF-induced heating, and heat-triggered DOX release for enhanced chemotherapy [226]; (**b**) FeAu@MIL-100(Fe) uses a FeAu alloy core and MOF shell to integrate magnetic hyperthermia, drug delivery, and imaging capability, enabling magnetically guided multimodal therapy against oral tumor models [227]; (**c**) PHIONs-AIPH employs Fe_3_O_4_-mediated magnetothermal conversion to trigger AIPH decomposition under AMF, producing cytotoxic free radicals for oxygen-independent tumor cell killing [228]; (**d**) MTDR combines magnetic nanoparticles with a thermoresponsive PNIPAM-grafted MOF structure, allowing AMF-induced heating to regulate drug release [229].

As shown in Figure 10, several MOF-based magnetothermal platforms have been developed to enhance tumor therapy. Meng et al. developed a MOF-derived Fe_3_O_4_@C nanocomposite by transforming an Fe-MOF precursor into a porous Fe_3_O_4_@C core-shell architecture under microwave-assisted synthesis, followed by PVP modification and DOX loading. The Fe_3_O_4_ core provided T_2_-weighted MRI contrast and magnetic responsiveness, while the porous carbon shell enabled drug loading and controlled DOX release. Under an AMF, the system generated magnetothermal heating and promoted drug release, thereby integrating MRI guidance, MHT, and CT for enhanced tumor cell killing [226]. Dhawan et al. reported a theranostic FeAu@MIL-100(Fe) platform by integrating FeAu alloy nanoparticles within a MIL-100(Fe)-type MOF shell. In this core-shell architecture, the FeAu core provided magnetic responsiveness and imaging capability, whereas the porous MOF shell functioned as a DOX reservoir for controlled CT. Upon exposure to an AMF, magnetothermal heating promoted DOX release, enabling imaging-guided MHT-CT. By combining a magnetic bimetallic core with a drug-loadable MOF shell, this platform integrated heat generation, controlled drug delivery, and diagnostic functions within a single nanotherapeutic system [227]. Huang et al. developed an AMF-responsive Fe_3_O_4_-based nanoplatform, PHIONs-AIPH, to achieve magnetothermal-triggered radical therapy. In this system, the water-soluble radical initiator AIPH was encapsulated within the porous carrier and further stabilized by a phase-change material coating. Upon exposure to an alternating magnetic field, Fe_3_O_4_-mediated magnetothermal heating triggered the decomposition of AIPH, generating cytotoxic free radicals. Because this radical-generation process does not rely on oxygen availability, PHIONs-AIPH enabled effective tumor cell killing even under hypoxic conditions, providing a representative strategy for coupling MHT with oxygen-independent radical therapy [228]. Ge et al. designed a magnetothermal-responsive drug release platform (MTDR) by integrating carboxylated Fe_3_O_4_ nanoparticles into a UiO-66-NH_2_ framework and grafting thermoresponsive PNIPAM onto the particle surface. The PNIPAM layer acted as a temperature-gated barrier that limited premature 5-FU leakage under physiological conditions. Upon alternating magnetic field exposure, Fe_3_O_4_-mediated heating induced PNIPAM collapse, rapidly opening the release pathway for 5-FU. This thermally gated release process enabled localized MHT to be synchronized with on-demand CT, thereby enhancing tumor cell killing [229]. Overall, these studies indicate that MOF-based architectures provide an effective framework for optimizing magnetic hyperthermia beyond the intrinsic heating performance of magnetic nanoparticles. By confining or organizing magnetic components within porous MOF structures, these platforms can improve particle dispersion, reduce aggregation, and integrate magnetothermal conversion with MRI contrast and drug delivery. Magnetothermal heating can also function as a remote activation signal, triggering phase-change materials, thermoresponsive polymers, or radical initiators to achieve on-demand release and oxygen-independent tumor killing under hypoxic conditions. Unlike PTT, MHT is not constrained by optical penetration depth. Its therapeutic precision is nevertheless strongly governed by the intratumoral distribution of magnetic components. Therefore, the major contribution of MOF-based systems in MHT lies in their capacity to control magnetic particle delivery, spatial organization, imaging traceability, and stimulus-responsive therapeutic release within an integrated nanoplatform.

In summary, PTT enables precise local heating with straightforward operational requirements, making it suitable for tumors where thermal damage is confined and easily controlled. MWH provides deeper tissue penetration and allows controlled local energy deposition, which is particularly advantageous for treating deep-seated tumors, though achieving uniform thermal fields and sufficient tumor selectivity remains a key challenge. MHT offers remote activation and the potential for concurrent imaging guidance, yet it demands careful design and spatial control of magnetic nanocomponents to ensure effective heating. MOF-based compounds, with their tunable porous structures, controllable metal nodes, and surface functionalization, allow structural and functional optimization for different energy modalities. This facilitates efficient thermal conversion and more uniform local temperature elevation, thereby enhancing the efficacy and safety of various hyperthermia strategies.

**Table 2:** Representative MOF-Based Platforms for Tumor Hyperthermia.

MOF Platform	Main Composition Or Structural Design	Heating Modality	Thermal Conversion Efficiency	Tumor Model	Therapeutic Outcome
Cu_10_MOF [184]	Cu metalated porphyrinic Zr MOF based on PCN	PTT	27.6%	4T1	Enhanced photothermal response and ROS generation
MnO_2_@C@TiO_2−x_ [187]	MIL 125 derived TiO_2−x_ carbon platform with MnO_2_ incorporation	PTT	36.4%	CT26	Improved photothermal conversion and ROS generation
CuS@Cu-MOF/PEG [191]	Cu MOF partially converted into CuS nanodot containing composite with PEG modification	PTT	47.9%	4T1	Cell viability decreased to 15.3% after 8 min NIR exposure
ICG CpG@MOF [194]	MIL-101 NH_2_ based MOF conjugated with ICG and loaded with CpG	PTT	33.7%	B16F10	Combined tumor cell killing and immune activation
CNP NO@RBCs [197]	ZIF 8 derived porous carbon nanoparticles loaded with L arginine and cloaked with RBC membrane	PTT	40.2%	4T1	Combined photothermal therapy and NO gas therapy
FCMP [210]	Fe Cu bimetallic organic framework modified with mPEG	MWH	32.5%	H22	Coupled microwave sensitization with ROS mediated CDT and GSH depletion
Fe Cu MOF@PEG@SUN [211]	Fe Cu bimetallic MOF loaded with sunitinib	MWH	35.8%	Hepg2	Suppressed primary and metastatic tumors by combining MWH with PD-L1 regulation
5-FUGR [212]	Ga MOF core cloaked with hybrid red blood cell and platelet membrane	MWH	38.1%	CT26	Improved tumor accumulation and promoted immunogenic cell death
FdMI [213]	Mn doped Zr MOF integrated with confined ionic liquids and fucoidan modification	MWH	41.3%	4T1	Improved local MW energy deposition and ROS amplified antitumor efficacy
Cu Zr MOF [214]	Cu doped Zr MOF MW sensitizer	MWH	36.7%	H22	Produced thermal elevation, ROS amplification, HSP70 downregulation, and ferroptosis related tumor cell death
DDS ZPDMP [215]	Janus nanorocket integrating ZIF-67 propulsion module with DOX loaded DMSNs	MWH	34.9%	Hela	Enhanced tumor penetration and intracellular drug accumulation
MOF-199@Shikonin@HA-PEG [216]	Cu based MOF-199 loaded with shikonin and modified with HA-PEG	MWH	39.5%	4T1	Glycolysis inhibition, ATP suppression, GSH depletion, and ROS amplification
CMALRH [217]	CoMnMOF cloaked with red blood cell and hyaluronic acid hybrid membrane	MWH	42.6%	CT26	Hypoxia relief and amplified ROS mediated therapeutic damage
GCZMT [218]	ZrMOF NH_2_ loaded with NO donor and HSP70 regulating agent, functionalized with TPP	MWH	44.1%	H22	Induced programmed cell death and immunogenic cell death
Fe_3_O_4_@C PVP@DOX [226]	MOF derived Fe_3_O_4_@C core shell nanocomposite loaded with DOX	MHT	28.6%	4T1	Integrated magnetothermal therapy with controlled CT
FeAu@MIL-100(Fe) [227]	FeAu alloy core integrated with MIL-100(Fe) MOF shell and DOX reservoir	MHT	41.2%	U14	Combined heat generation, controlled drug delivery, and diagnostic function
PHIONs AIPH [227]	Fe_3_O_4_ based porous nanoplatform encapsulating AIPH and stabilized by phase change material	MHT	36.5%	B16F10	Generated oxygen independent cytotoxic radicals for hypoxic tumor killing
MTDR [229]	Carboxylated Fe_3_O_4_ nanoparticles integrated into UiO-66 NH_2_ and grafted with PNIPAM	MHT	43.8%	4T1	Achieved temperature gated drug release and enhanced tumor cell killing

## 4. Integrated Strategies for MOF-Based Hyperthermia

MOF-based materials can substantially enhance energy conversion within tumor regions, yet thermotherapy alone often fails to overcome tumor heterogeneity, thermotolerance, and immunosuppression [230]. This limitation is particularly pronounced in mild thermotherapy or treatment of deep-seated tumors, where sublethal thermal exposure allows residual tumor cells to activate heat shock proteins and antioxidant defense systems, promoting survival and contributing to recurrence or metastasis [231]. MOF-based hyperthermia platforms offer particular advantages for multimodal therapy not simply because they can co-load multiple therapeutic components, but because their programmable architectures enable spatial integration of heat generation, drug delivery, catalytic reactions, oxygen regulation, immune modulation, and imaging within a single nanosystem. Unlike physical mixtures of photothermal agents, chemotherapeutic drugs, or catalytic components, the metal nodes, organic ligands, pore structures, and surface chemistry of MOFs can jointly regulate drug ratios, release sites, and activation timing, allowing different therapeutic processes to be coupled within the TME.

Hyperthermia exerts dependent effects on ROS-associated therapies. Local temperature elevation can accelerate molecular diffusion, increase membrane permeability, promote drug release, and enhance the kinetics of Fe, Cu, or Mn-mediated Fenton and Fenton-like reactions, thereby increasing the generation of highly reactive species such as ·OH [232]. Heating may also weaken antioxidant defenses and sensitize tumor cells to oxidative damage. However, sustained hyperthermia can increase cellular metabolism and oxygen consumption, and may further aggravate local hypoxia through vascular damage or reduced perfusion [233]. In transition-metal MOFs, Fe^2+^/Fe^3+^, Cu^+^/Cu^2+^, and Mn^2+^/Mn^3+^/Mn^4+^ redox cycling can convert endogenous H_2_O_2_ into cytotoxic ·OH, while hyperthermia can accelerate reaction kinetics and facilitate metal-ion release, thereby amplifying oxidative stress [234]. True therapeutic synergy should therefore involve more than parallel cytotoxic effects; heat should enhance catalytic efficiency, disrupt antioxidant barriers, or promote regulated cell death pathways in ways that are mechanistically connected to the companion therapy. The interaction between hyperthermia and IT is likewise temperature- and sequence-dependent. Moderate heating can promote tumor antigen release, DAMP exposure, and immunogenic cell death, thereby supporting dendritic cell maturation, T-cell priming, and immune infiltration into tumors [235]. MOF platforms can further extend this local response by delivering immune adjuvants, immune checkpoint inhibitors, or other immunomodulatory agents, potentially converting localized thermal injury into a broader antitumor immune response. Excessive or premature heating, however, may induce vascular shutdown, restrict immune-cell trafficking, and intensify hypoxia, which can compromise immune activation. Treatment sequence is therefore a critical design variable in MOF-based combination therapy, determining whether heat generation, ROS amplification, drug release, oxygen modulation, and immune stimulation occur in a mutually reinforcing manner or merely as independent additive effects.

To address these challenges, MOF-based thermotherapeutic platforms are increasingly designed to combine thermal effects with additional therapeutic mechanisms. The porous framework, tunable metal nodes, and surface functionalization of MOFs enable the co-delivery of chemotherapeutic drugs, photosensitizers, catalytic centers, metabolic modulators, and immunotherapeutic agents. Such multifunctional platforms can simultaneously induce thermal injury, oxidative stress, metabolic disruption, immune activation, and facilitate imaging-guided monitoring [236]. The purpose of combination therapy is not simply to stack multiple functional modules within a single platform, but to use mechanistic synergy between different therapeutic modalities to address key limitations that arise during hyperthermia. CT can eliminate residual cells that survive sublethal thermal injury, CDT can amplify heat-induced oxidative stress, metabolic therapy (MT) can reduce the energy supply required for HSP synthesis and cellular repair, and IT can convert local thermal damage into systemic antitumor immune responses. Therefore, rational MOF-based combination platforms should be constructed around clearly defined therapeutic mechanisms, rather than through the indiscriminate addition of functional components. In this context, combination strategies integrating hyperthermia with CT, CDT, MT, and IT are emphasized to achieve mechanistic complementarity and enhanced antitumor efficacy.

### 4.1. CT-Hyperthermia Strategy

The combination of hyperthermia and CT represents a foundational and widely employed synergistic strategy in MOF-based cancer treatment. Thermal stimulation enhances drug accumulation in tumor tissues by increasing cell membrane fluidity and vascular permeability, while elevated temperatures accelerate MOF framework degradation and promote drug diffusion, ensuring synchronized drug release and heat exposure [237]. Chemotherapeutic agents subsequently eliminate tumor cells that survive sublethal thermal stress, reducing the risk of recurrence [238]. Compared with conventional free drugs, MOF-based delivery systems improve local drug retention and enable tumor microenvironment-responsive release, thereby increasing tumor selectivity [239].

**Figure 11:**
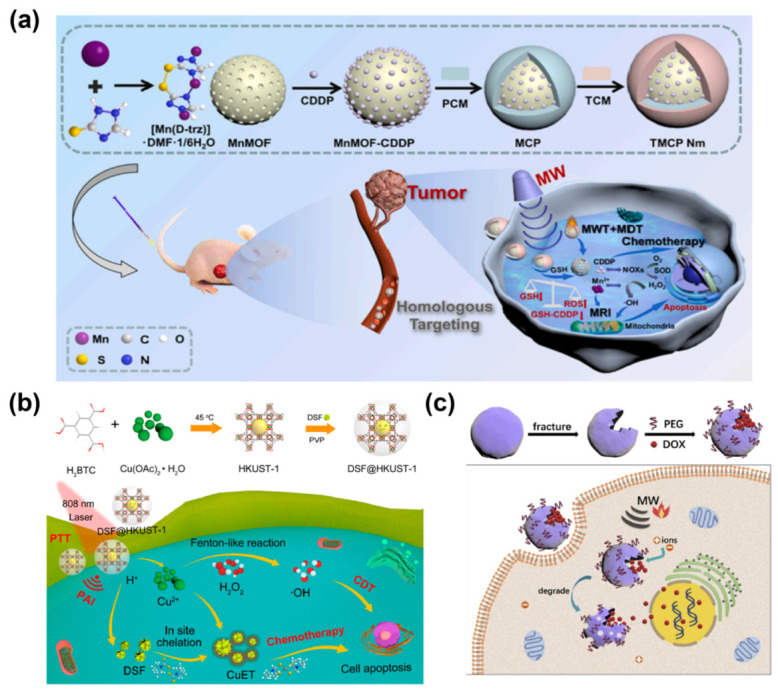
Representative MOF-based platforms for hyperthermia-chemotherapy combination therapy. (**a**) MnMOF-CDDP@PCM-TCM integrated cisplatin-loaded MnMOF with a phase-change material and tumor cell membrane coating, enabling thermally responsive drug release, tumor-targeted delivery, and enhanced efficacy [241]; (**b**) DFS@HKUST-1 underwent degradation in the acidic TME, releasing Cu^2+^ to coordinate with DSF and form the CuET complex for in situ CT, accompanied by enhanced photothermal effects [242]; (**c**) Construction of DOX@ZDNP@PEG by loading DOX into Zr-MOF-derived open-mouthed nano-popcorns followed by PEG surface modification for combined MWH and CT [243].

The synergy between hyperthermia and CT extends beyond additive effects. Thermal stress sensitizes tumor cells to DNA-damaging agents, topoisomerase inhibitors, and mitochondria-targeting drugs, while chemotherapeutic agents induce apoptosis, DNA lesions, or mitochondrial dysfunction, further compromising the cellular thermal resistance [240]. This bidirectional enhancement achieves cooperative effects at both molecular and cellular levels. As shown in Figure 11, Wu et al. designed a MnMOF-CDDP@PCM-TCM nanomissile by encapsulating cisplatin (CDDP) within a manganese-based MOF, followed by phase-change material coating and tumor cell membrane camouflage. The MnMOF framework served as a microwave-responsive carrier, whereas the PCM layer enabled thermally regulated drug release and the TCM coating improved tumor-targeted accumulation. Under MW irradiation, localized heating promoted CDDP release and simultaneously impaired ATP production in cisplatin-resistant tumor cells, thereby reducing drug efflux and increasing intracellular drug retention. This strategy coupled MWH with CT to overcome drug resistance and enhance tumor cell killing [241]. Similarly, Shen et al. developed DFS@HKUST-1 by incorporating disulfiram (DSF) into the Cu-based MOF HKUST-1, followed by PVP surface modification, to achieve TME-responsive, photoacoustic imaging-guided PTT-CT. In the acidic TME, HKUST-1 underwent framework degradation and released Cu^2+^, which reacted with DSF in situ to generate the cytotoxic CuET complex. The Cu-based framework also contributed to local heat generation, enabling photothermal ablation under external irradiation. Importantly, thermal stimulation further accelerated MOF degradation and drug release, rapidly increasing intracellular CuET formation and enhancing the elimination of tumor cells that survived sublethal heat stress. This system therefore achieved temporally and spatially coordinated PTT-CT through acid-triggered activation, heat-enhanced release, and imaging-guided intervention [242]. Li et al. developed Zr-MOF-derived open-mouthed nano-popcorns (ZDNPs) using a sonochemical aerosol flow strategy, and further engineered them with DOX loading and PEG surface modification to obtain DOX@ZDNP@PEG for combined MWH and CT. The open-cracked architecture of ZDNPs enhanced ion confinement and collision under microwave irradiation, resulting in higher microwave-to-heat conversion efficiency than conventional Zr-MOF. In parallel, the crack-rich porous structure enabled DOX loading and pH-responsive release under acidic conditions. In vivo results showed that DOX@ZDNP@PEG combined with MW irradiation markedly increased intratumoral temperature and suppressed tumor growth, highlighting the potential of MOF-derived MW sensitizers for synergistic MWH-CT [243].

### 4.2. CDT-Hyperthermia Strategy

The integration of hyperthermia and CDT represents a typical strategy for exploiting the multifunctionality of MOF-based tumor platforms. CDT relies on metal-catalyzed Fenton or Fenton-like reactions to convert endogenous H_2_O_2_ in the TME into highly reactive radicals, especially ·OH, thereby inducing oxidative stress-mediated tumor cell damage [244]. However, the efficacy of CDT is often restricted by insufficient H_2_O_2_ availability, hypoxia, limited catalytic efficiency, and strong intracellular antioxidant defenses, including GSH and GPX4. Hyperthermia can help overcome these limitations by accelerating catalytic reactions, improving mass transport, and weakening cellular redox homeostasis [245].

**Figure 12:**
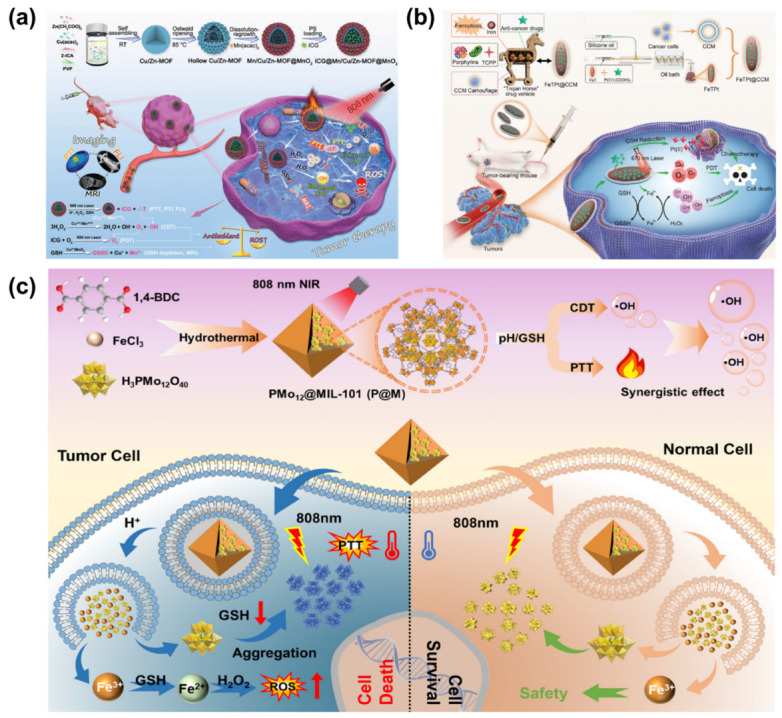
(**a**) ICG@Mn/Cu/Zn-MOF@MnO_2_ combines ICG-mediated PTT with Mn/Cu-driven Fenton-like reactions. NIR irradiation induces local heat generation, while dual-metal catalysis promotes ROS production, GSH depletion, and tumor cell damage [249]; (**b**) FeTPt@CCM MOF uses cancer cell membrane camouflage to enhance homologous tumor targeting and accumulation. After cellular uptake, the Fe/Pt-based framework promotes CDT, hyperthermia-associated damage, and ferroptosis-related tumor cell death [250]; (**c**) PMo12@MIL-101 responds to acidic and GSH-rich tumor microenvironments to activate Fe^2+^-mediated ·OH production. Under NIR irradiation, photothermal heating further amplifies CDT, resulting in selective tumor cell killing while reducing damage to normal cells [251].

Elevated temperature promotes metal ion mobility, electron transfer, and substrate collision, thereby accelerating Fenton-like reactions and enhancing ROS generation [246]. Moderate heating can also increase membrane permeability and facilitate the diffusion of H_2_O_2_, O_2_, and therapeutic agents into tumor cells or MOF pores. Meanwhile, heat-induced mitochondrial dysfunction increases endogenous ROS production, which further amplifies oxidative stress together with ROS [247]. Conversely, excessive ROS can impair heat-resistance and antioxidant defense pathways, including HSPs, GSH, and GPX4, thereby lowering the threshold for heat-induced cellular injury [248]. Thus, hyperthermia and CDT can reinforce each other through coupled thermal and oxidative damage. As shown in Figure 12, Cheng et al. reported a multimetallic hollow MOF-derived nanocomposite, ICG@Mn/Cu/Zn-MOF@MnO_2_, for photothermal-enhanced CDT. The Cu/Zn-MOF precursor was converted into a hollow porous framework and further engineered with Mn^2+^/MnO_2_ to establish a multivalent catalytic system. In the TME, Cu^+^ and Mn^2+^ promoted Fenton-like ·OH generation from endogenous H_2_O_2_, whereas Cu^2+^ and MnO_2_ consumed intracellular GSH, thereby weakening the antioxidant defense of tumor cells. Upon NIR irradiation, ICG-mediated photothermal heating increased the local temperature and further accelerated ·OH production, strengthening CDT-mediated oxidative damage. By integrating multimetallic redox cycling, GSH depletion, and heat-amplified ROS generation, this platform achieved synergistic photothermal-chemodynamic antitumor activity [249]. Zhang et al. fabricated a cancer cell membrane-coated FeTPt@CCM MOF nanoplatform using a microfluidic strategy, integrating Fe^3+^ nodes, the photosensitizer TCPP, and a cisplatin prodrug within a single FeTPt framework. The CCM coating endowed the nanoparticles with homologous tumor-targeting capability and enhanced cellular internalization. After endocytosis, the Fe-containing framework could participate in chemodynamic reactions, while the TCPP and platinum prodrug components supported light-triggered therapy and CDT, respectively. This biomimetic MOF design enabled tumor-targeted delivery and multimodal antitumor activity through the coordinated integration of PTT and CDT [250]. Li et al. reported a GSH/pH-responsive PMo_12_@MIL-101 nanoplatform by confining redox-activatable PMo_12_ clusters within the porous MIL-101 framework for combined PTT and CDT. The MIL-101 host improved the stability and cellular uptake of PMo_12_, while enabling acidic TME-triggered framework disassembly and the release of both PMo_12_ and Fe^3+^. In tumor cells, GSH converted PMo_12_ into a NIR-absorbing photothermal species and reduced Fe^3+^ to Fe^2+^, thereby coupling NIR-triggered heat generation with Fenton-mediated ·OH production from H_2_O_2_. The resulting local hyperthermia further accelerated the Fe^2+^-mediated Fenton reaction, leading to amplified ROS production and enhanced tumor cell killing [251].

### 4.3. Metabolic Therapy-Hyperthermia Strategy

In recent years, tumor hyperthermia has shifted from solely relying on direct thermal ablation to targeting adaptive survival mechanisms that emerge following sublethal heating. Tumor cells exposed to thermal stress can repair damage by enhancing glycolysis, increasing ATP production, upregulating HSPs, and activating antioxidant defenses. Such adaptive responses may allow residual tumor cells to survive, recur, or acquire greater invasive potential [252]. Combining hyperthermia with metabolic interventions has therefore emerged as an effective strategy to improve long-term outcomes by disrupting tumor energy supply and heat resistance pathways.

**Figure 13:**
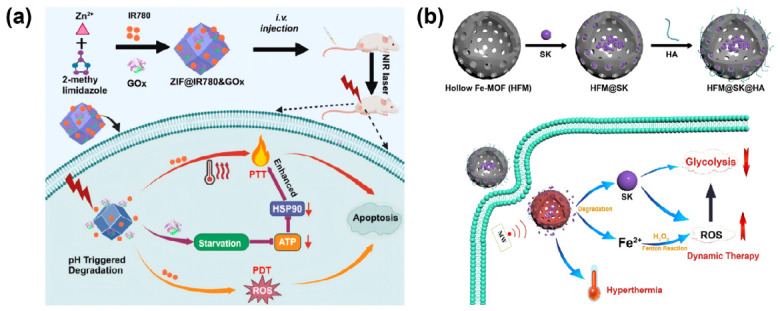
(**a**) ZIF-8@IR780@GOx integrates GOx-mediated glucose depletion with photothermal therapy, suppressing ATP production and attenuating thermotolerance to sensitize tumor cells to photothermal injury [254]; (**b**) HFM@SK@HA enables microwave-responsive shikonin release, thereby inhibiting glycolysis, weakening heat resistance, and enhancing oxidative stress through intracellular ROS generation [255].

Metabolic interventions aim to reduce ATP generation, thereby suppressing HSP synthesis, impairing the repair of misfolded proteins, and disrupting membrane homeostasis [253]. Common approaches include the use of GOx to deplete glucose and glycolysis inhibitors such as shikonin to reduce energy generation, thereby increasing tumor sensitivity to thermal stimulation. As shown in Figure 13, Qi et al. engineered a pH-responsive ZIF-8@IR780@GOx (ZIG) nanoplatform through a one-pot assembly strategy, integrating the NIR-responsive IR780 and glucose oxidase within a degradable ZIF-8 framework. Under acidic tumor conditions, ZIF-8 gradually disassembled and enabled localized release of IR780 and GOx. The released GOx catalyzed glucose oxidation to generate gluconic acid and H_2_O_2_, thereby depleting glucose-derived energy supply and reducing ATP availability. This metabolic stress downregulated HSP90 expression and weakened the ability of tumor cells to tolerate photothermal injury. By combining IR780-mediated thermal damage with GOx-induced metabolic inhibition, ZIG produced a synergistic enhancement of PTT [254]. Similarly, Chen et al. developed a tumor-targeted hollow Fe-MOF nanoplatform (HFM@SK@HA), by loading shikonin into the MOF cavity and decorating the surface with hyaluronic acid. Under MW irradiation, the hollow Fe-MOF enabled controlled shikonin release, allowing glycolytic inhibition to occur in parallel with microwave-induced heating. By suppressing tumor glycolysis, shikonin reduced energy availability and weakened thermotolerance, thereby increasing tumor susceptibility to thermal damage. Meanwhile, the Fe-based framework promoted intracellular ROS generation and oxidative stress, further amplifying microwave-mediated tumor killing. This strategy links metabolic interference, redox disruption, and MWH within a tumor-targeted MOF platform [255]. Metabolic intervention is highly complementary to hyperthermia because thermal stress imposes a substantial bioenergetic demand on tumor cells. After heat exposure, ATP-dependent processes such as protein refolding, membrane repair, ion homeostasis, and antioxidant defense are required for cellular recovery and thermotolerance. Disrupting energy metabolism can therefore sensitize tumor cells to thermal injury. GOx-mediated glucose depletion or glycolysis inhibition can restrict ATP production, suppress HSP-associated stress responses, and impair repair pathways, thereby reducing the likelihood of recovery after sublethal heating. MOF platforms are well suited for this therapeutic logic, as they can integrate metabolic regulators with photothermal or microwave-responsive components and enable localized activation through acidic TME conditions or external energy stimulation. This makes MOF-based systems attractive candidates for coupling hyperthermia with metabolic vulnerability to achieve more durable antitumor effects.

**Figure 14:**
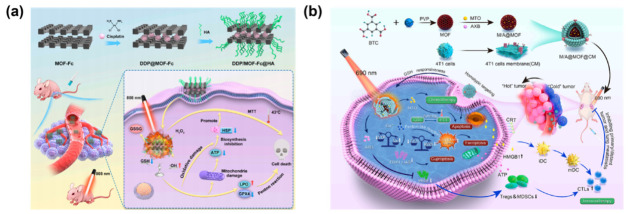
(**a**) DDP/MOF-Fc@HA integrates cisplatin (DDP) delivery with Fc MOF-mediated Fenton catalysis, enabling mild photothermal therapy combined with amplified ferroptosis [257]; (**b**) M/A@MOF@CM releases Cu^2+^ in response to tumor microenvironmental GSH, disrupting redox balance and copper homeostasis, thereby inducing cuproptosis through targets such as DLAT [259].

The integration of thermotherapy with metabolism-associated regulated cell death offers another route to strengthen antitumor efficacy. Heat stress can disturb membrane organization and mitochondrial homeostasis, thereby increasing oxidative stress and lipid peroxidation. These changes provide a favorable basis for ferroptosis, an iron-dependent form of regulated cell death characterized by excessive lipid peroxide accumulation and membrane damage [256]. In turn, ferroptosis can impair mitochondrial function, amplify ROS production, suppress heat-protective responses, and reduce the capacity of tumor cells to recover from sublethal thermal injury. Deng et al. engineered an HA-modified Fc-based MOF nanoplatform, DDP/MOF-Fc@HA, for mild PTT coupled with ferroptosis amplification. The Fc-containing framework served both as a photothermal component and as a Fenton-active catalytic source, producing local hyperthermia under NIR irradiation and converting endogenous H_2_O_2_ into ·OH within the TME. This heat-enhanced ROS generation promoted lipid oxidative damage and ferroptotic cell death. In parallel, the loaded cisplatin (DDP) depleted intracellular GSH, weakened antioxidant buffering, and downregulated key ferroptosis-suppressive proteins, including GPX4, SLC7A11, and frataxin. Through the convergence of mild thermal stress, Fenton catalysis, GSH depletion, and ferroptosis pathway regulation, this platform enhanced the antitumor efficacy of mild PTT [257] (Figure 14a).

Similarly, cuproptosis has also been explored as a complementary cell death pathway in MOF-based thermotherapeutic systems. This copper-dependent form of regulated cell death is associated with disturbed copper homeostasis, mitochondrial metabolism, and protein lipoylation. In Cu-based MOFs, the release of Cu^2+^ can enhance tumor cell killing through copper toxicity, GSH depletion, and Fenton-like catalytic reactions [258]. Ji et al. developed a cancer cell membrane-camouflaged Cu-MOF nanoplatform, M/A@MOF@CM, by co-loading mitoxantrone (MTO) and the anti-VEGF agent AXB for homologous tumor-targeted delivery. In the GSH-rich tumor microenvironment, framework degradation promoted the release of Cu^2+^ and the encapsulated therapeutic agents. The liberated Cu^2+^ contributed to redox disruption through Fenton-like reactions and further disturbed intracellular copper homeostasis. This Cu-dependent stress was associated with cuproptosis-related mechanisms, including interactions with lipoylated mitochondrial targets such as DLAT, thereby providing an additional apoptotic route for tumor cell killing [259] (Figure 14b). Ferroptosis and cuproptosis introduce additional mechanistic dimensions for improving the sensitivity of tumors to hyperthermia. Unlike apoptosis-centered strategies, these regulated cell death pathways are tightly linked to metal ion metabolism, mitochondrial dysfunction, lipid peroxidation, and collapse of antioxidant buffering systems. Thermal stress can increase membrane susceptibility to oxidative lipid damage and aggravate mitochondrial vulnerability, thereby lowering the threshold for non-apoptotic cell death. In this context, Fe or Cu-based MOFs are particularly attractive because their released metal ions can drive Fenton or Fenton-like reactions, deplete GSH, disrupt copper homeostasis, and amplify oxidative or metabolic stress. Such coupling enables transient heat-induced injury to be converted into more persistent ferroptotic or cuproptotic tumor cell death.

### 4.4. IT-Hyperthermia Strategy

The combination of thermotherapy and IT leverages local heating to convert tumors into in situ antigen sources, inducing immunogenic cell death (ICD) and promoting dendritic cell maturation and effector T-cell infiltration [260]. Thermotherapy can stimulate tumor cells to release damage-associated molecular patterns (DAMPs), including calreticulin (CRT) exposure, HMGB1 release, and HSP expression. These signals enhance antigen uptake, antigen presentation, and the initiation of adaptive immune responses [261]. Previous studies have shown that CRT can promote the phagocytosis of tumor cells by DCs, whereas HMGB1 can enhance antigen presentation through the TLR4 pathway, thereby initiating a complete cancer-immunity cycle [262].

**Figure 15:**
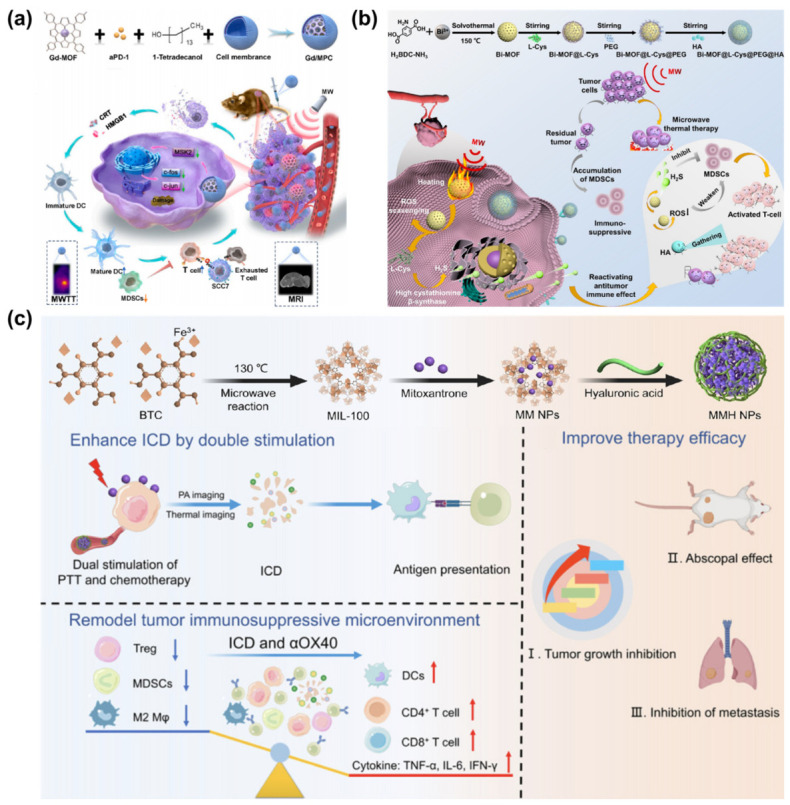
(**a**) Gd/MPC encapsulated aPD-1 through microfluidics, promoted DC maturation and antigen presentation, and activated CD4^+^ and CD8^+^ T cells, thereby achieving synergistic thermotherapy and IT [265]; (**b**) BMCPH responded to the TME by releasing L-cysteine, generating H_2_S, reversing immunosuppression, and enhancing systemic antitumor immune responses [266]; (**c**) MIL-100-based MMH nanoparticles combine MTO-mediated CT and laser-triggered PTT to induce ICD, and further synergize with αOX40 immunostimulation to remodel the immunosuppressive TME [267].

However, thermotherapy alone is often insufficient to overcome the immunosuppressive TME shaped by regulatory T cells, myeloid-derived suppressor cells, tumor-associated macrophages, and PD-L1 overexpression [263]. MOF-based platforms can synchronize heat-induced antigen release with immunomodulatory interventions, such as immune checkpoint blockade, adjuvants, or IDO inhibition [264]. For instance, Gd-MOF@aPD-1@CM (Gd/MPC) was developed as a microwave-responsive immunotherapeutic platform by incorporating aPD-1 into a Gd-based MOF, followed by phase-change material integration and cancer cell membrane camouflage. The cancer cell membrane enhanced homologous tumor targeting, whereas microwave-induced heating triggered controlled aPD-1 release and local tumor ablation. This treatment induced immunogenic cell death, characterized by CRT exposure, HMGB1 release, and ATP secretion, and further promoted dendritic cell maturation and CD4^+^/CD8^+^ T-cell activation. By coupling MWH with checkpoint blockade, Gd/MPC enabled synergistic tumor thermotherapy and IT [265] (Figure 15a). Similarly, Bi-MOF@L-Cys@PEG@HA (BMCPH) was designed to integrate MWH with tumor-responsive H_2_S-mediated immunomodulation. In this platform, Bi-MOF served as the microwave-responsive thermal component, while L-cysteine provided a tumor-activated source of H_2_S. Under MW irradiation, BMCPH generated localized hyperthermia and increased tumor susceptibility to thermal damage. Concurrently, L-cysteine-derived H_2_S alleviated the immunosuppressive TME by reducing ROS-associated stress and suppressing MDSC accumulation. This immune remodeling promoted CD8^+^ T-cell activation and tumor infiltration, thereby converting local MW heating into a stronger systemic antitumor immune response [266] (Figure 15b). Ni et al. designed a MIL-100-based MMH nanoplatform by encapsulating MTO and modifying the particle surface with hyaluronic acid. In this system, MTO served not only as a chemotherapeutic agent but also as a photothermal component, enabling laser-triggered heat generation within the tumor. The combination of MTO-mediated CT and photothermal heating markedly enhanced immunogenic cell death, accompanied by increased CRT exposure, HMGB1 release, and ATP secretion. When this local treatment was combined with αOX40 immunostimulation, the therapeutic response was further amplified through enhanced dendritic cell activation, increased CD4^+^ and CD8^+^ T-cell infiltration, and reduced accumulation of immunosuppressive MDSCs, M2 macrophages, and Tregs. In vivo results showed that MMH nanoparticles combined with laser irradiation and αOX40 not only significantly suppressed primary tumor growth, but also induced distant tumor inhibition and reduced lung metastasis [267] (Figure 15c).

From the perspective of the cancer-immunity cycle, hyperthermia-immunotherapy requires more than local tumor destruction; it must support a cascade of immune events, including antigen release, antigen presentation, T-cell priming, tumor infiltration, and effector-mediated killing. Hyperthermia primarily initiates this cascade by promoting tumor antigen liberation and DAMP exposure, thereby creating conditions favorable for immunogenic cell death. However, the subsequent immune response, is frequently constrained by immunosuppressive features of the tumor microenvironment, such as PD-L1 upregulation, MDSC expansion, Treg activity, and TAM polarization. MOF-based platforms offer a means to connect thermal tumor damage with downstream immune activation by incorporating checkpoint inhibitors, immunoadjuvants, or TME-remodeling agents into hyperthermia systems. Such integration helps convert localized thermal stress into systemic antitumor immunity, thereby improving the therapeutic reach of MOF-mediated hyperthermia.

## 5. Intelligent Stimuli-Responsive MOF-Based Hyperthermia Platforms

### 5.1. TME-Responsive Platforms

The TME exhibits distinct characteristics relative to normal tissues, providing a natural basis for precise and targeted thermotherapy. Typical features of the TME include acidic pH, high osmotic pressure, elevated levels of H_2_O_2_, high concentrations of GSH, and disordered oxygen metabolism [268]. The acidic environment can trigger the degradation of the MOF framework, thereby promoting the controlled release of drugs or functional molecules. In addition, the high GSH level and reducing conditions can induce electron transfer or dissociation of variable-valence metal nodes within MOFs, leading to the responsive release of metal ions or ligands and thus enabling microenvironment-driven precise therapy [269].

TME-responsive MOF platforms are designed to achieve selective therapeutic activation by capitalizing on the biochemical differences between tumor and normal tissues, rather than depending exclusively on passive tumor accumulation. The acidic TME can destabilize metal-ligand coordination and promote framework disassembly, whereas elevated intracellular GSH can cleave redox-sensitive linkages or reduce variable-valence metal nodes, leading to the controlled release of therapeutic drugs and catalytic ions. Abundant H_2_O_2_ and hypoxia can also be leveraged to support catalytic ROS generation, oxygen production, or redox modulation. When integrated with externally applied hyperthermia, these endogenous triggers enable MOF platforms to follow a staged therapeutic process, beginning with tumor enrichment, followed by microenvironment-specific activation, and culminating in localized release or catalytic amplification. Moreover, the highly vascularized and irregular blood flow network in tumors further facilitates MOF accumulation via the enhanced permeability and retention (EPR) effect. The tunable particle size, porous architecture, and surface functionalization enhance retention and uniform distribution at tumor sites [270]. Because the EPR effect is highly heterogeneous across tumor types and patient populations, passive accumulation alone rarely provides sufficient reliability for hyperthermia-based therapy. MOF platforms are therefore frequently engineered to improve systemic circulation, tumor retention, and intratumoral penetration through size control, hydrophilic surface modification, biomimetic membrane camouflage, and ligand-mediated targeting. Cancer cell membrane coating can promote homologous tumor recognition, hyaluronic acid can facilitate CD44-mediated uptake, and folate or peptide ligands can enhance receptor-specific internalization. By increasing the intratumoral concentration of hyperthermia agents, these strategies improve the spatial selectivity of external energy deposition and help minimize nonspecific thermal damage to surrounding normal tissues.

**Figure 16:**
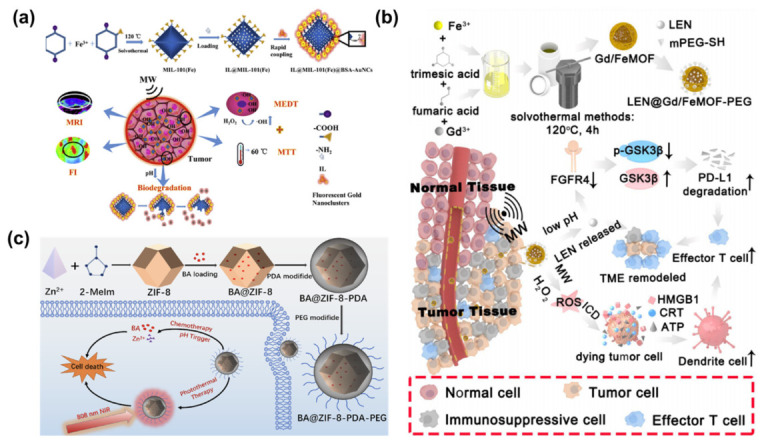
(**a**) IL@MIL-101(Fe)@BSA-AuNCs incorporated ILs within the MOF framework and decorated the surface with BSA-Au nanoclusters, providing MW responsiveness, biodegradability, and dual-modal imaging functionality [271]; (**b**) LEN@Gd/FeMOF-PEG integrated Gd and Fe to achieve T1-T2 dual-modal MRI for precise tumor localization and simultaneously enhanced ROS generation under MWH [272]; (**c**) BA@ZIF-8-PDA-PEG enables acidic TME-responsive BA release and PDA-mediated photothermal heating for enhanced chemo-photothermal tumor therapy [273].

As shown in Figure 16, Ma et al. developed a multifunctional iron-based metal-organic framework nanozyme, IL@MIL-101(Fe)@BSA-AuNCs, by loading ionic liquids (ILs) into MIL-101(Fe) nanoparticles and decorating the surface with BSA-Au nanoclusters. This design endowed the system with MW responsiveness, biodegradability, and dual-modal imaging capability. Taking advantage of the acidic conditions and elevated H_2_O_2_ levels in the tumor microenvironment, the system generated ROS through the peroxidase-like activity of Fe^3+^/Fe^2+^, thereby inducing tumor cell death. The nanoparticles could also degrade in response to the TME, releasing Fe^3+^ and maintaining local activity, which helped preserve therapeutic efficacy while reducing systemic toxicity [271]. Wang et al. developed a lenvatinib-loaded Gd/Fe bimetallic MOF nanoplatform, LEN@Gd/FeMOF-PEG, to enhance the antitumor immune response induced by MW thermotherapy. The incorporation of Gd and Fe not only provided T1-T2 dual-modal MRI capability for accurate tumor localization, but also enhanced ROS generation during MW thermotherapy. In the tumor microenvironment, Gd/FeMOF efficiently catalyzed the generation of ·OH and ^1^O_2_ under MW irradiation, thereby inducing ICD. In addition, the acidic TME accelerated MOF degradation and promoted the release of lenvatinib. By inhibiting the FGFR4-GSK3β signaling pathway, lenvatinib reduced PD-L1 expression and further reversed the immunosuppressive TME. This effect reduced the infiltration of MDSCs, Tregs, and M2-type TAMs, while increasing the number of CD8^+^ T cells and the levels of Th1 cytokines, including IL-2 and IFN-γ [272]. Gao et al. designed BA@ZIF-8-PDA-PEG (BZPP) as a tumor microenvironment-responsive chemo-photothermal nanoplatform. In this system, the natural antitumor compound baicalein (BA) was incorporated into pH-sensitive ZIF-8, followed by PDA coating and surface modification with PEG. The acid-labile ZIF-8 framework enabled selective BA release in the acidic tumor microenvironment, whereas the PDA shell served as the NIR-responsive photothermal component. Under laser irradiation, BZPP showed concentration and power-dependent heating behavior. The surface modification with PEG further improved colloidal stability, hemocompatibility, and overall biosafety. In A549 cells, BZPP achieved efficient cellular uptake and enhanced tumor cell killing under NIR irradiation through the combined effects of BA-mediated CT and PDA-mediated PTT [273].

### 5.2. Image-Guided Hyperthermia

Image guidance has become an essential element in the development of MOF-based thermotherapy toward precision cancer treatment. By enabling real-time visualization of nanoplatform biodistribution and treatment progression, multimodal imaging improves the safety, controllability, and efficacy of thermotherapy. The structural diversity of MOF-based materials makes them particularly suitable for imaging integration. Metal nodes such as Mn, Fe, Gd, Cu, Bi, or Zr can contribute to magnetic resonance imaging (MRI), computed tomography, or photoacoustic imaging, while organic ligands, loaded dyes, or surface probes can introduce fluorescence and thermal imaging functions. As a result, MOF-based thermotherapeutic systems can support image-guided tumor localization before treatment, real-time monitoring during energy application, and post-treatment assessment of therapeutic response [274].

Before thermotherapy, imaging can confirm whether the nanoplatform has accumulated sufficiently within the tumor, thereby helping to define the optimal timing for laser irradiation, microwave exposure, or magnetic-field activation [275]. For instance, MOFs containing Mn or Fe-based nodes can act as MRI contrast agents and provide T1 or T2-weighted signal enhancement, allowing high-contrast visualization of material distribution in vivo [276]. Computed tomography and fluorescence imaging can also provide visual information based on differences in metal density or the incorporation of fluorescent probes. During treatment, image-guided monitoring is important for controlling heat deposition and reducing off-target injury [277]. Infrared thermal imaging and MR thermometry can track temperature changes in real time, helping to avoid both overheating of normal tissues and insufficient heating of tumor regions [278]. Photoacoustic imaging can additionally provide information on oxygenation and blood perfusion, which is useful for evaluating the dynamic response of the TME and adjusting treatment parameters accordingly [279]. After treatment, MRI, computed tomography, fluorescence imaging, and photoacoustic imaging can be used to follow nanomaterial clearance, tumor necrosis, residual lesions, and recurrence, thereby extending the role of imaging from procedural guidance to longitudinal therapeutic evaluation [280]. The integration of multimodal imaging not only improves the precision and safety of hyperthermia, but also provides a basis for early detection of recurrence and secondary intervention.

**Figure 17:**
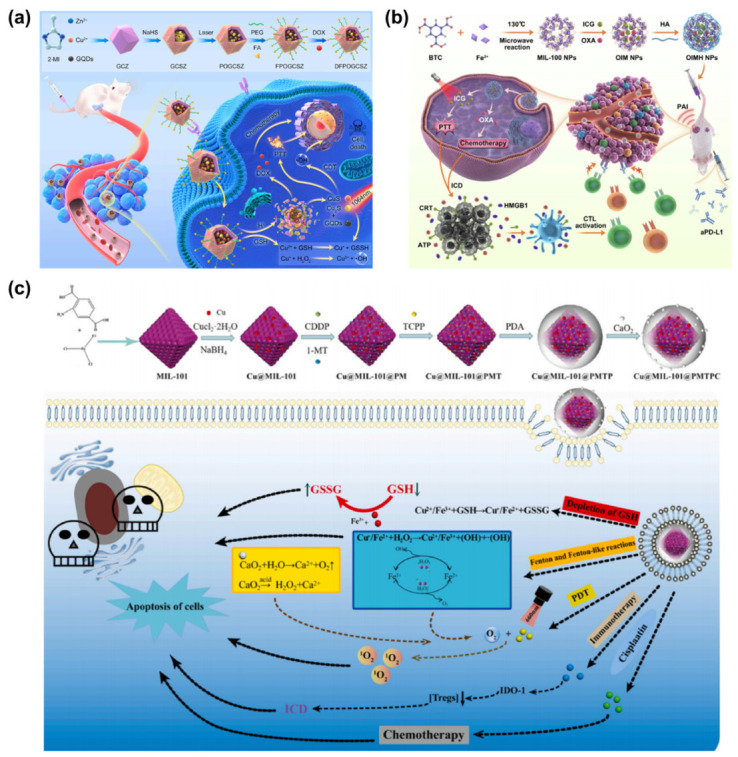
(**a**) GCSZ/POGCSZ combined PAI, FLI, and MRI to monitor in vivo nanoparticle distribution and drug release, enabling precise hyperthermia [281]; (**b**) HA-modified MIL-100(Fe) co-delivers OXA and ICG to enable photoacoustic imaging-guided chemo-photothermal therapy, with PA signal feedback guiding the laser irradiation window [282]; (**c**) Cu@MIL-101@PMTPC incorporated the photosensitizer TCPP, chemotherapeutic agent Pt, and immunomodulator 1-MT to achieve photothermal responsiveness, tumor targeting, and multimodal therapy, while providing MRI, fluorescence, and photoacoustic imaging for treatment guidance [283].

As shown in Figure 17, Wang et al. designed an eggshell-like Cu_2_S@ZIF-8 nanoplatform doped with graphene quantum dots (GQDs), termed GCSZ/POGCSZ, for imaging-guided PTT-CT. The Cu_2_S core provided strong NIR photothermal performance, while the ZIF-8 shell enabled DOX loading and pH-responsive release in the acidic TME. Partial surface oxidation further generated CuS, strengthening optical absorption and photothermal conversion. Folic acid modification conferred active tumor-targeting capability. Notably, the incorporation of GQDs and Cu-based components endowed the system with fluorescence/photoacoustic imaging and MRI functions, enabling in vivo visualization of nanoparticle accumulation and drug release. This multimodal imaging feedback supported optimization of the irradiation window and improved the spatiotemporal control of PTT [281]. Liu et al. designed an OIMH nanoplatform by integrating oxaliplatin (OXA) and indocyanine green (ICG) within a hyaluronic acid-modified MIL-100(Fe) framework for imaging-guided chemo-photothermal therapy. MIL-100(Fe) served as a porous host for the co-delivery of OXA and ICG, while ICG endowed the system with both NIR-triggered photothermal activity and photoacoustic imaging capability. The HA modification further improved stability and facilitated tumor-targeted cellular uptake. Upon laser irradiation, OIMH generated efficient and stable photothermal heating in a concentration-dependent manner. In vivo photoacoustic imaging showed gradual accumulation of OIMH at the tumor site, with the strongest PA signal observed at 12 h after injection, thereby providing a rational time window for laser irradiation [282]. An et al. developed Cu@MIL-101@PMTPC as a multifunctional MIL-101(Fe)-based nanoplatform for imaging-guided multimodal therapy. Ultrasmall Cu nanoparticles were integrated onto the MOF surface, while TCPP, Pt, and 1-MT were incorporated to provide photodynamic, chemotherapeutic, and immunomodulatory functions. Further modification with dopamine and CaO_2_ endowed the platform with photothermal activity, oxygen-generating capability, and tumor-responsive release behavior. In addition to these therapeutic modules, Cu@MIL-101@PMTPC enabled MRI, fluorescence imaging, and photoacoustic imaging, allowing dynamic visualization of nanoparticle distribution and therapeutic progression [283]. This theranostic design illustrates how MOF platforms coordinate photothermal activation, oxygen modulation, drug delivery, and multimodal imaging to improve the precision of combination therapy.

## 6. Challenges and Prospects

Despite the rapid progress of MOF-based platforms for tumor hyperthermia, the field remains predominantly at the proof-of-concept stage. Most reported systems have been validated in cell models and animal tumor models, whereas evidence supporting clinical feasibility is still limited. A major challenge arises from the increasing complexity of multifunctional nanoplatforms. Although the integration of heat generation, drug delivery, catalytic therapy, immune modulation, and imaging can improve therapeutic efficacy in experimental settings, excessive structural complexity may compromise synthetic reproducibility, quality control, regulatory evaluation, and clinical safety. Future MOF-based hyperthermia systems should therefore move from function stacking toward mechanism-oriented design. Rather than incorporating as many therapeutic modules as possible, platform construction should prioritize essential functions, clear therapeutic logic, and controllable structure–function relationships.

Although MOF-based materials can improve energy conversion and enhance local antitumor responses, the biological constraints of tumor hyperthermia remain substantial. Intratumoral heterogeneity in vascular density, perfusion, stromal composition, oxygenation, and material distribution can result in highly uneven heat deposition. Consequently, some tumor regions may reach therapeutically effective temperatures, whereas well-perfused areas or regions with insufficient energy absorption may remain within a sublethal thermal range, allowing residual tumor cells to survive, repair damage, and contribute to recurrence [284]. For PTT, NIR-I irradiation offers operational convenience and efficient photothermal activation, but its limited tissue penetration and strong attenuation by skin, blood, and tissue scattering make homogeneous heating difficult in deep-seated or large solid tumors [285]. MWH and MHT provide greater tissue penetration potential, yet their thermal profiles are still influenced by tissue dielectric properties, blood perfusion, magnetic material distribution, and external field parameters, which may lead to central overheating, insufficient marginal heating, or collateral injury to adjacent normal tissues [286]. The biological consequences of hyperthermia are highly dependent on temperature and exposure duration. High-temperature thermal ablation can induce vascular shutdown, coagulative necrosis, and rapid tissue destruction, but vascular collapse may also restrict subsequent oxygen supply, drug delivery, and immune-cell infiltration, thereby compromising downstream combination therapy [287]. Mild hyperthermia can improve perfusion, increase drug penetration, and promote immunogenic cell death, but its direct cytotoxicity is relatively limited. When the delivered thermal dose is insufficient, tumor cells may develop thermotolerance through heat shock protein upregulation, enhanced antioxidant defense, and mitochondrial functional recovery [288]. Oxygen dynamics during heating are also context-dependent. Transient temperature elevation may temporarily improve perfusion and oxygenation, whereas sustained or excessive heating can damage tumor vasculature, increase oxygen consumption, and exacerbate hypoxia, thereby affecting ROS-dependent therapy, immune-cell infiltration, and post-treatment recurrence risk. The TME and immune contexture further determine whether local thermal injury can be translated into durable systemic antitumor immunity. Hyperthermia may induce tumor antigen release, DAMP exposure, and immunogenic cell death, but immunosuppressive factors such as Tregs, MDSCs, tumor-associated macrophages, and PD-L1 upregulation can limit T-cell activation and infiltration [289]. Thus, increasing local temperature alone does not necessarily produce sustained antitumor immunity.

Despite their advantages in energy conversion, drug delivery, and multimodal therapy, MOF-based materials still face substantial toxicity and long-term biosafety challenges that limit clinical translation. Metal nodes may dissociate in acidic TME, lysosomes, or complex physiological media, leading to the release of ions such as Fe, Cu, and Mn. Controlled local ion release can support Fenton-like catalysis, GSH depletion, MRI contrast enhancement, or regulated cell death. However, excessive or systemic ion leakage may cause nonspecific oxidative stress, mitochondrial dysfunction, hepatic and renal burden, neurotoxicity, and other forms of systemic toxicity [290]. Future studies should therefore distinguish therapeutic local metal-ion release from potentially harmful systemic leakage, and improve the safety window through coordination-stability regulation, surface shielding, and stimulus-responsive degradation. The long-term biodistribution, metabolism, and clearance pathways of MOF nanoparticles also remain insufficiently defined. Highly stable or relatively large MOF particles may persist in the liver, spleen, and reticuloendothelial system, increasing the risk of chronic inflammation, tissue fibrosis, and organ dysfunction. Accordingly, short-term tumor inhibition data and H&E staining of major organs are not sufficient to establish biosafety. More rigorous evaluation should include long-term pharmacokinetics, biodegradation profiles, blood biochemistry, and safety assessment after repeated administration [291]. Surface modification and biomimetic coating can improve circulation stability and tumor targeting, but they may also alter protein corona formation, complement activation, cytokine release, and immune recognition after repeated dosing. Their immunogenicity, hemocompatibility, and long-term inflammatory responses therefore require systematic assessment [292]. Future MOF-based hyperthermia platforms should not be designed solely to maximize thermal conversion efficiency or increase therapeutic complexity. Establishing standardized biosafety evaluation frameworks will be essential for advancing MOF-based materials from proof-of-concept studies toward preclinical validation and eventual clinical translation.

From a translational perspective, MOF-based materials remain at a stage between laboratory proof of concept and preclinical validation. Their clinical applicability will depend on whether they can achieve stable tumor accumulation, controllable heat generation, and predictable therapeutic responses in complex biological environments. Compared with single-component photothermal agents or conventional inorganic nanomaterials, MOFs offer a programmable structural framework that can integrate thermal conversion, drug delivery, TME modulation, and imaging monitoring, thereby providing a potential basis for image-guided and individualized hyperthermia. However, clinical translation will require further progress in scalable synthesis, batch-to-batch reproducibility, and quality control. Particle size, morphology, metal-node ratio, pore architecture, drug-loading capacity, and surface modification can all influence biodistribution and therapeutic performance. Standardized synthetic protocols, critical quality attribute assessment, and reproducible scale-up manufacturing processes are therefore necessary. Regulatory considerations represent another major barrier. MOF-based hyperthermia platforms often combine features of drugs, devices, and diagnostic agents, and may therefore fall within regulatory pathways for combination products. The long-term degradation behavior, metal-ion release, immunogenicity, and compatibility with external energy devices all require systematic evaluation. From a commercialization perspective, MOF platforms with clearly defined indications, simplified composition, controllable manufacturing processes, and compatibility with existing clinical hyperthermia equipment are more likely to be developed further. Future design should therefore move beyond functional stacking and instead prioritize clinical usability and manufacturability. MOF-based hyperthermia systems with well-defined composition, verifiable biosafety, quantifiable treatment parameters, and integrated imaging guidance or therapeutic monitoring will have greater translational relevance. In addition, precise energy deposition and thermal dose control remain major barriers to MOF-mediated hyperthermia. Although photothermal therapy offers high spatial controllability, its efficacy is limited by shallow optical penetration, particularly for deep-seated tumors. Microwave and magnetic hyperthermia provide greater penetration depth, but still suffer from heterogeneous heat distribution, insufficient marginal heating, central overheating, and potential injury to adjacent normal tissues. These challenges cannot be addressed by material optimization alone. Future development should integrate MOF engineering with energy-delivery strategies, including thermal-field simulation, real-time temperature monitoring, and image-guided treatment planning. Standardized assessment frameworks are essential for improving cross-platform comparability and translational relevance in MOF-based hyperthermia research. At present, substantial variation in material dose, administration route, tumor model, energy source, irradiation parameters, magnetic-field settings, and thermal measurement methods makes it difficult to distinguish genuine material advantages from differences in experimental design. Future studies should report key performance metrics in a more consistent manner, including energy conversion efficiency, tissue penetration depth, and systemic immune activation. For combination therapy platforms, appropriate control groups are required to define the contribution of each therapeutic module, and claims of synergy should be supported by mechanistic evidence rather than tumor growth inhibition alone.

Advances in artificial intelligence, machine learning, and computational modeling are increasingly reshaping the rational design of MOF-based nanomedicine. MOF-based hyperthermia platforms involve multiple interdependent variables, including metal nodes, organic ligands, pore architecture, surface chemistry, drug-loading strategy, and responsiveness to external energy fields. Conventional trial-and-error screening is often time-consuming and costly, and it provides limited insight into the complex relationships between structural parameters and therapeutic performance. By integrating MOF structural databases with experimentally derived performance datasets, machine learning models could be used to predict framework stability, pore characteristics, drug-loading capacity, thermal conversion behavior, and biocompatibility, thereby enabling the prescreening of candidate materials before experimental synthesis [293]. For tumor hyperthermia, computational modeling can further support the prediction of energy conversion, local thermal distribution, and tissue temperature evolution under different external fields, providing a basis for optimizing treatment parameters and thermal dose control [294]. Molecular dynamics simulations, density functional theory calculations, and multiphysics modeling may also help elucidate electron transfer between metal nodes and organic ligands, defect-mediated energy dissipation, and non-radiative relaxation pathways, thereby guiding mechanism-informed MOF design [295]. In this context, artificial intelligence and computational modeling are expected to move MOF-based materials beyond empirical optimization toward data-driven and mechanism-guided development, supporting the construction of intelligent therapeutic platforms with more defined composition, predictable performance, and assessable safety profiles.

Overall, the development of MOF-based tumor hyperthermia should transition from the pursuit of increasingly complex multifunctional architectures to mechanism-oriented, structurally defined, and clinically feasible systems. A balanced integration of efficient energy conversion, selective tumor localization, and biosafety will be essential for advancing MOF-based compounds from experimental hyperthermia enhancers to clinically relevant platforms for precise and personalized cancer therapy.

## 7. Conclusions

As highly tunable porous organic–inorganic hybrid materials, MOF-based compounds provide structurally tunable platforms for tumor hyperthermia, but their practical value depends on reproducible synthesis, controllable degradation, thermal dose regulation, and long-term biosafety. Across photothermal, microwave, and magnetic hyperthermia, MOF-based systems have demonstrated unique advantages in energy conversion, thermal regulation, and multimodal therapeutic integration. Beyond heat generation, MOF-based platforms have expanded the therapeutic scope of hyperthermia. The high surface area and modifiable pore structures of MOFs enable the loading of chemotherapeutic drugs, photosensitizers, catalytic metal centers, metabolic regulators, and immune modulators. As a result, MOF-based hyperthermia can be combined with chemotherapy, chemodynamic therapy, metabolic intervention, ferroptosis, cuproptosis, immunotherapy, and multimodal imaging. These combinations are particularly valuable for overcoming the limitations of heat monotherapy, including thermotolerance, recurrence, incomplete tumor ablation, and insufficient immune activation. Importantly, MOF-based compounds are evolving from passive nanocarriers into active therapeutic systems capable of microenvironment-responsive regulation, cell death induction, immune modulation, and imaging-guided intervention.

However, the clinical translation of MOF-based hyperthermia is still hindered by several challenges, including long-term biosafety concerns, metal ion leakage, and manufacturing scalability. Future efforts should focus on improving biocompatibility, thermal precision, and intelligent responsive design to accelerate the development of precise, personalized, and clinically translatable hyperthermia-based cancer therapies.

## Data Availability

No new data were created or analyzed in this study.
